# The HOPX and BLBP landscape and gliogenic regions in developing human brain

**DOI:** 10.1111/joa.13844

**Published:** 2023-02-16

**Authors:** Camilla Bjørnbak Holst, Christian Beltoft Brøchner, Kristoffer Vitting‐Seerup, Kjeld Møllgård

**Affiliations:** ^1^ Department of Cellular and Molecular Medicine, Faculty of Health and Medical Sciences University of Copenhagen Copenhagen Denmark; ^2^ DCCC Brain Tumor Center Copenhagen University Hospital Copenhagen Denmark; ^3^ Department of Pathology, Center of Diagnostic Investigation Copenhagen University Hospital Copenhagen Denmark; ^4^ Section for Bioinformatics, Health Technology Technical University of Denmark (DTU) Denmark

**Keywords:** BLBP, CNS, fetal, HOPX, human

## Abstract

Outer radial glial cells (oRGs) give rise to neurons and glial cells and contribute to cell migration and expansion in developing neocortex. HOPX has been described as a marker of oRGs and possible actor in glioblastomas. Recent years' evidence points to spatiotemporal differences in brain development which may have implications for the classification of cell types in the central nervous system and understanding of a range of neurological diseases. Using the Human Embryonic/Fetal Biobank, Institute of Cellular and Molecular Medicine, Faculty of Health and Medical Sciences, University of Copenhagen, Denmark, HOPX and BLBP immunoexpression was investigated in developing frontal, parietal, temporal and occipital human neocortex, other cortical areas and brain stem regions to interrogate oRG and HOPX regional heterogeneity. Furthermore, usage of high‐plex spatial profiling (Nanostring GeoMx^®^ DSP) was tested on the same material. HOPX marked oRGs in several human developing brain regions as well as cells in known gliogenic areas but did not completely overlap with BLBP or GFAP. Interestingly, limbic structures (e.g. olfactory bulb, indusium griseum, entorhinal cortex, fimbria) showed more intense HOPX immunoreactivity than adjacent neocortex and in cerebellum and brain stem, HOPX and BLBP seemed to stain different cell populations in cerebellar cortex and corpus pontobulbare. DSP screening of corresponding regions indicated differences in cell type composition, vessel density and presence of apolipoproteins within and across regions and thereby confirming the importance of acknowledging time and place in developmental neuroscience.

## Graphical abstract

HOPX is not only a marker for outer radial glial cells but also is present throughout human developing brain in various cell types and structures. Multiplex spatial profiling may give new insights into spatiotemporal heterogeneity in brain development.

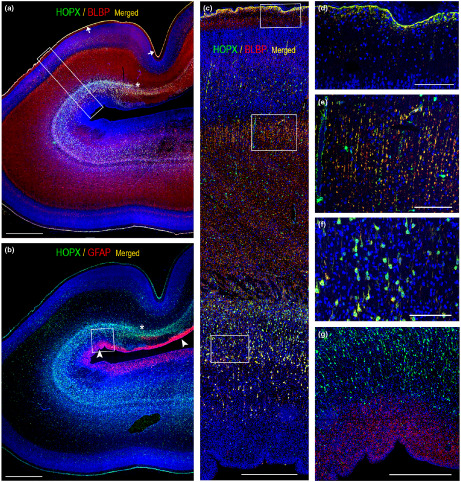

## 1 INTRODUCTION

Homeodomain‐only protein (HOPX) is an atypical homeodomain protein lacking DNA binding sites (Zweifel et al., 2018) and known as a marker of outer radial glial cells (oRGs) including their basal fibers (Pollen et al., 2015). Radial glial cells are neural stem cells located in the ventricular (VZ) and subventricular zone (SVZ) of the developing neocortex (Pollen et al., 2015) and crucial for cell migration and lamination (Borrell & Götz, 2014). Classical radial glial cells, located in the VZ, anchor apically at the ventricular surface and extend with radial fibers to the pial surface or terminate with endfeet on the walls of blood vessels (Holst et al., 2019; Schmechel & Rakic, 1979). Apical truncated radial glia cells have lost their pial connection, whereas outer radial glial cells retain their basal endfeet at the pial surface or on blood vessels but lose their apical contact and mainly reside in the outer subventricular zone (OSVZ) (Pollen et al., 2015). Outer radial glial cells are believed to generate the majority of upper cortical layer neurons, neurons in other cortical layers (Molnár et al., 2019; Pollen et al., 2015; Smart et al., 2002), and contribute to gliogenesis (Pollen et al., 2015; Zhong et al., 2020), migration (Nowakowski et al., 2016) and gyrification in developing primate brain (Matsumoto et al., 2020; Rash et al., 2019), although the latter is still a matter of debate. Considering proportions of oRGs found in lissencephalic mouse medial neocortex (Vaid et al., 2018) and near‐lissencephalic primate SVZ (García‐Moreno et al., 2012; Kelava et al., 2012), oRGs may be necessary but not sufficient for gyrencephaly (García‐Moreno et al., 2012) and the role of HOPX in cortical folding is still not fully determined (Matsumoto et al., 2020; Vaid et al., 2018). Given their function, neurodevelopmental diseases caused by defects in neurogenesis and neuronal migration have been associated with radial glial cells (Barry et al., 2014; Subramanian et al., 2020). Glioblastomas (GBM) are devastating lethal primary tumors of the central nervous system believed to arise from transformation of glial precursors or neural stem cells (Kim et al., 2020). HOPX has been proposed to be part of a gene panel characterizing bipotent fetal glial progenitor cells with unspecific localization in telencephalon and similar glioblastoma (GBM) cells (Couturier et al., 2020). In the mouse postnatal SVZ, dorso‐medial cells have been found to mainly express HOPX and astrocytic markers whereas lateral and dorso‐lateral cells are enriched with transcripts of neuronal markers (Zweifel et al., 2018). HOPX also label cortical astrocytes (Falcone et al., 2021) and HOPX‐expressing neural stem cells in postnatal mouse brain are biased to generate astrocytes (Zweifel et al., 2018), establishing HOPX as a marker of the astroglial lineage and possible player in gliomas. However, HOPX does not seem to have a direct role in astrocyte specification (Zweifel et al., 2018).

Laminar organization of the developing neocortex in primates has primarily been defined through studies of specific areas of the telencephalic wall (Altman & Bayer, 2002; Kostović et al., 2002, 2019; Smart et al., 2002) and the general use of these terms is based on the notion of cortical uniformity. However, comparisons between neocortical areas reveal several differences in zonal structure (Altman & Bayer, 2002; Kostović et al., 2019) (Table 1), gene expression in individual cell types (Bhaduri et al., 2021) as well as earlier maturation of rostral telencephalic wall (Bystron et al., 2008; Nowakowski et al., 2017) and distinct neuronal progeny (Nowakowski et al., 2017). Expression patterns of radial glial cells from the prefrontal cortex and primary visual cortex intermingle across multiple clusters in single‐cell mRNA analysis, indicating that radial glial cells follow a typological hierarchy, but also show accelerated maturation of frontal cortex radial glia (Nowakowski et al., 2017). In other cortical regions, as well as in the brain stem, zones may be organized differently and are less extensively studied. In developing hippocampus, HOPX+ progenitors may have different cell fates depending on their location (Zhong et al., 2020) and single‐cell RNA sequencing analysis of developing human brain indicates that gene expression differs across all cell types comparing different brain regions (Bhaduri et al., 2021; Zhong et al., 2020) with stronger regionalization in glial populations (Bhaduri et al., 2021).

**TABLE 1:** Laminar organization of developing neocortex.

	(Smart et al., 2002)	(Altman & Bayer, 2002)	(Altman & Bayer, 2002)	(Kostović et al., 2002)	(Bystron et al., 2008)	(Žunić Išasegi et al., 2018)			(Žunić Išasegi et al., 2018)	(Pollen et al., 2015)
Species	Monkey	Human	Human	Human		Human			Human	Human
Cortical region	Occipital	Parietal/occipital (granular)	Frontal (agranular)	Parietal/occipital	Ventrolateral part of dorsal telencephalon	Parietal/occipital			Frontal	
Age	E65‐E94^a^	13.5–20 gw^b^	13.5–20 gw^b^	15–29 wpc^c^	14 gw^b^	21–28 wpc			21 wpc	14–16 gw^b^
Pia	**MZ**	SGL	SGL	MZ	SGL	MZ				MZ
					MZ					
	**CP** (↑)	CP	CP	CP	CP	CP			CP	CP
	**SP** (↑)^g^	*SP*	SP	SP^4,6^	SP	SP			SP	SP
		STF1^3^	STF1^3^ (↑)							
		*STF2* ^d^ (↑)	STF2 (↑)							
	**OFL** ^**1,2**^	STF3^e^ ^,4^ (trilaminar structure)	Absent	IZ^7,4,8,5^	IZ	External transient proliferative cell band (8)		MACC	MACC (zones less strictly defined than in occipital lobe)	IZ
						ESS^7,4^ (7)				
						Outer proliferative cell layer (6)	OSVZ			
	**OSVZ** (E65‐E72 ↑) (E88‐E94 ↓)	*STF4* ^4^	STF4^f^,^1^		SVZ	ISS (5)				SVZ
		*STF5* (multilaminar matrix)	STF5 (↓) (thin perikaryal layer)	SVCZ		Inner proliferative cell layer (4)				
	**IFL** ^h^	*STF6*	STF6^5^	PVFZ					PVFZ^5^ (callosal septa)	
	**ISVZ**	SVZ	SVZ	VZ					ISVZ	
Ventricle	**VZ** (↓)	NEP	NEP		VZ	VZ (1)			VZ	VZ

*Note*: Bold: nomenclature used in present paper; Italics: poorly developed/less prominent/more narrow; ↑/↓: Change in size over time; Fiber composition: ^1^corticofugal fibers; ^2^corticopetal including geniculostriate fibers; ^3^early component of white matter, with the difference that cells are still migrating through STF1; ^4^thalamocortical fibers; ^5^callosal fibers; ^6^cortico‐cortical fibers; ^7^external capsule fibers; ^8^cortical efferent fibers.

Abbreviations: CP, cortical plate; ESS, external sagittal stratum; ISS, internal sagittal stratum; IZ, intermediate zone; MACC, multilaminar axonal‐cell compartment; MZ, marginal zone; NEP, neuroepithelium; PVFZ, periventricular fibre rich zone; SGL, subpial granular layer; SP, subplate; STF, stratified transitional field; STF1, the upper fibrous layer; STF2, the upper perikaryal layer; STF3, the middle multilaminar matrix (trilaminar honeycomb matrix); STF4, the middle fibrous layer (continuous with the internal capsule); STF5, the lower multilaminar matrix; STF6, the lower fibrous layer (continuous with the corpus callosum); SVCZ, subventricular cellular zone; VZ, ventricular zone.

^a^ E = days after fertilization, E78 monkey corresponds to 19 wpc human (www.translatingtime.org).

^b^ wpc = gw ‐ 2 w.

^c^ Original nomenclature: post ovulatory weeks = wpc.

^d^ In the sagittal plane STF2 is inconspicuous in the occipital lobe.

^e^ The trilaminar structure of STF3 is most prominent in occipital cortex.

^f^ Thalamocortical fibers may also be present in the paracentral lobule.

^g^ Cellular increase from E65‐E72; decrease in packing density from E72‐E78.

^h^ Layer complete by E78.

Given the impact of outer radial glial cells on brain development and astrogliogenesis and an increasing evidence of regional heterogeneity in developing and adult brains, we interrogated spatiotemporal immunoexpression of HOPX, as well as BLBP (Brain Lipid‐Binding Protein), a radial glial cell marker (Feng et al., 1994), in developing frontal, parietal, temporal and occipital human neocortex, other cortical areas and brain stem regions around midgestation as a continuation of our work exploring spatiotemporal complexity in astrogliogenesis (Holst et al., 2019). Furthermore, we tested applicability of high‐plex spatial profiling (Nanostring GeoMx^®^) on the same fetal specimens expanding the usage of this unique material.

## 2 MATERIALS AND METHODS

### 2.1 Tissue samples

Brains from embryos and fetuses (5–200 mm CRL) corresponding to 5–21 wpc (weeks post conception) from legal abortions were obtained and examined after informed consent from all contributing women following oral and written information, in accordance with the Helsinki Declaration II, and approved by the Danish Regional Committee on Health Research Ethics (KF–V.100.1735/90) & (KF‐112006–4838). Major attention was paid to two mid‐term fetal brains (19 wpc and 21 wpc) due to the completeness of samples covering the entire brain and the initiation of gliogenesis.

The embryos and fetuses are part of the Human Embryonic/Fetal Biobank, Institute of Cellular and Molecular Medicine, Faculty of Health and Medical Sciences, University of Copenhagen, Denmark, as previously described in detail (Holst et al., 2019).

Post‐operative treatment of tissue consisted of immediate dissection of the samples into blocks and their fixation for 12–24 h at 4°C in either 10% neutral buffered formalin, 4% Formol‐Calcium, Lillie's or Bouin's fixatives. For immunohistochemistry, 2–10 μm thick serial sections were cut in transverse, sagittal or horizontal planes, and placed on silanized glass slides.

### 2.2 Immunohistochemistry

For bright field light microscopy, sections were deparaffinized and rehydrated in xylene and ethanol following standard protocols. Some primary antibodies required heat‐induced antigen retrieval. Endogenous peroxidase was quenched using a 0.5% solution of hydrogen peroxide in TRIS buffered saline (TBS, 5 mM Tris–HCl, 146 mM NaCl, pH 7.6) for 15 min. Following rinses with TBS, non‐specific binding was inhibited by incubation for 30 min with blocking buffer (ChemMate antibody diluent S2022, DakoCytomation, Glostrup, Denmark) or 10% goat serum (BI‐04‐009‐1A, In Vitro) at room temperature prior to overnight incubation with primary antibodies at 4°C diluted in blocking buffer followed by a rinse with TBS. The REAL EnVision Detection System (Peroxidase/DAB+ rabbit/mouse, code K5007, DakoCytomation, Glostrup, Denmark) was used for detecting mouse and rabbit primary antibodies. The sections were washed with TBS, followed by incubation for 10 min with 3,3′‐diamino‐benzidine chromogen solution. Positive staining was recognized as a brown color. The sections were counterstained with Mayers hematoxylin (Ampliqon Laboratory Reagents, AMPQ00253.5000), dehydrated in graded alcohols and coverslipped with Pertex mounting medium (HistoLab, 00801).

For immunofluorescence, sections were prepared as for bright field light microscopy. For double labeling, overnight incubation at 4°C was performed either as a mix of primary antibodies or sequentially. Secondary fluorophore conjugated antibody(ies) was then added for 30 min at room temperature with Labelled Polymer–HRP anti‐mouse or anti‐rabbit (DakoCytomation, EnVision™+ System/HRP K4007 or K4003) followed by Tyramide Signal Amplification with either Alexa Fluor 488 (Invitrogen, Molecular Probes, T20912) or Alexa Fluor 594 (Invitrogen, Molecular Probes, T20925) for 7 min at room temperature. Subsequently, the sections were incubated for 30 min at room temperature with either biotin‐SP‐conjugated F(ab′)_2_ fragment donkey anti‐rabbit antibodies (Jackson ImmunoResearch, 711–066‐152) followed by streptavidin‐conjugated DyLight 594 (Vector Laboratories, SA5594), or with Alexa Fluor 488 goat anti‐mouse (Jackson ImmunoResearch, 115‐545‐166) or Dylight 594 goat anti‐mouse (Jackson ImmunoResearch, 115‐515‐145). Finally, a nuclear counterstain with DAPI (4′,6‐diamidino‐2‐phenylindole, Invitrogen, Molecular Probes, D1306) was added for 3 min, before sections were coverslipped.

Details of the primary and secondary antibodies including dilutions and suppliers are listed in Table 2. Control sections were incubated with mouse IgG1 or irrelevant rabbit antibodies, as well as subjected to omission of primary or secondary antibodies. These were always blank. In addition, the repeated staining patterns derived from this material have been similar over several years.

**TABLE 2:** Primary and secondary antibodies.

Primary antibodies	Host IgG	Dilution	HIER	Producer	Code number
HOPX	Rabbit IgG	1:200	Citrate buffer pH 6	Sigma Life Science	HPA030180
GFAP	Mouse IgG1k	1:50	Citrate buffer pH 6	Dako	M0761
Vimentin	Mouse IgG1, Kappa	1:100	Citrate buffer pH 6	Dako	M0725
BLBP	Mouse IgG2b	1:500–1:1000	‐/Citrate buffer pH 6	OriGene Technologies	AM09059PU‐S

Secondary antibodies	Host IgG	Dilution	Target	Producer	Code number
HOPX					
Biotin‐SP	Donkey IgG	1:200	Anti‐rabbit	Jackson	711‐066‐152
Dylight 594 Strept.		1:200		Vector	SA5594
Dylight 488	Goat	1:100	Anti‐rabbit	Jackson	111‐485‐144
EnVision+ System‐HRP labelled Polymer			Anti‐rabbit	Dako	K4003
TSA Alexa Fluor 488 tyramide		1:100		Molecular probes by life technologies	T20912
GFAP					
EnVision Polymer‐HRP			Anti‐mouse	Dako	K4007
TSA Alexa Fluor 594 tyramide		1:100		Molecular probes by life technologies	T20925
Dylight 594	Goat	1:100	Anit mouse	Jackson	115‐515‐146
Vimentin					
EnVision Polymer‐HRP			Anti‐mouse	Dako	K4007
TSA Alexa Fluor 488 tyramide		1:100		Molecular probes by life technologies	T20912
BLBP					
EnVision Polymer‐HRP			Anti‐mouse	Dako	K4007
TSA Alexa Fluor 594 tyramide		1:100		Molecular probes by life technologies	T20925
Dylight 594	Goat	1:100	Anit mouse	Jackson	115‐515‐146

Fluorescent full slide scans of selected double immunolabeled sections were acquired using a Zeiss Axio Scan.Z1 slide scanner with a 20×/0.8 Plan‐Apochromat objective. For laser scanning confocal microscopy a Zeiss LSM 710 or Zeiss LSM 780 Confocal Microscope was used with 20×/0.8 Plan‐Apochromat, 20×/0.8 Plan‐Neofluar or 40×/1.3 Oil Plan Neofluar objectives. Slide scan images and confocal images were analyzed, and individual optical sections were exported as TIFF files using Zeiss ZEN Blue Edition. Representative images chosen for figure editing were processed in Adobe Photoshop 22.2.0.

### 2.3 Nanostring GeoMx ^®^ digital spatial profiling (protein)

GeoMx^®^ Digital Spatial Profiling (DSP) of five tissue samples of human fetal brain (frontal cortical wall, 13 wpc; parietal cortical wall, 19 wpc; temporal cortical wall, 19 wpc; occipital cortical wall, 21 wpc and brain stem and cerebellum, 21 wpc) was performed through the Nanostring GeoMx^®^ DSP Technology Access Program. Antibodies coupled to photocleavable oliogonucleotide DSP tags were used to measure protein abundance of panels of proteins in selected regions of interest (ROIs). In short, slides were initially stained with GFAP (Glial fibrillary acidic protein) and Syto13 DNA dye to visualize tissue morphology guiding the selection of 12 geometric Regions of Interest (ROIs) per sample (Figure S1–S5; Table S1). Three fixed protein panels (Neural Cell Profiling Core Module, AD Pathology Module, PD Pathology Module) were then measured in each ROI. The negative control antibodies Ms IgG1, Ms IgG2a and Rbt IgG were used to assess background levels. The Nanostring nCounter^®^ was used to quantify the DSP tags and thus spatial protein expression levels within the respective ROIs. In this assay, hybridization positive controls (ERCC) were spiked into the assay to control for any technical assay variability during quantification. ERCC scaled data for each ROI were normalized using a scaling approach (average of geometric mean of Ms IgG1 and Rbt IgG for all ROIs divided by the geometric mean of Ms IgG1 and Rbt IgG for each selected ROI). ERCC normalized data were used to calculate log2 signal‐to‐noise ratios (SNR) using Ms IgG1, Ms IgG2a and Rbt IgG as negative background controls. SNR above 2 or log2 SNR above 1 was considered detectible signal. In unscaled heatmaps for selected proteins and areas/regions (Figure 5) color indicate log2 SNR and values below 1 or missing are depicted in grey.

## 3 RESULTS AND DISCUSSION

### 3.1 HOPX and radial glial cells in occipital cortex

**FIGURE 1:**
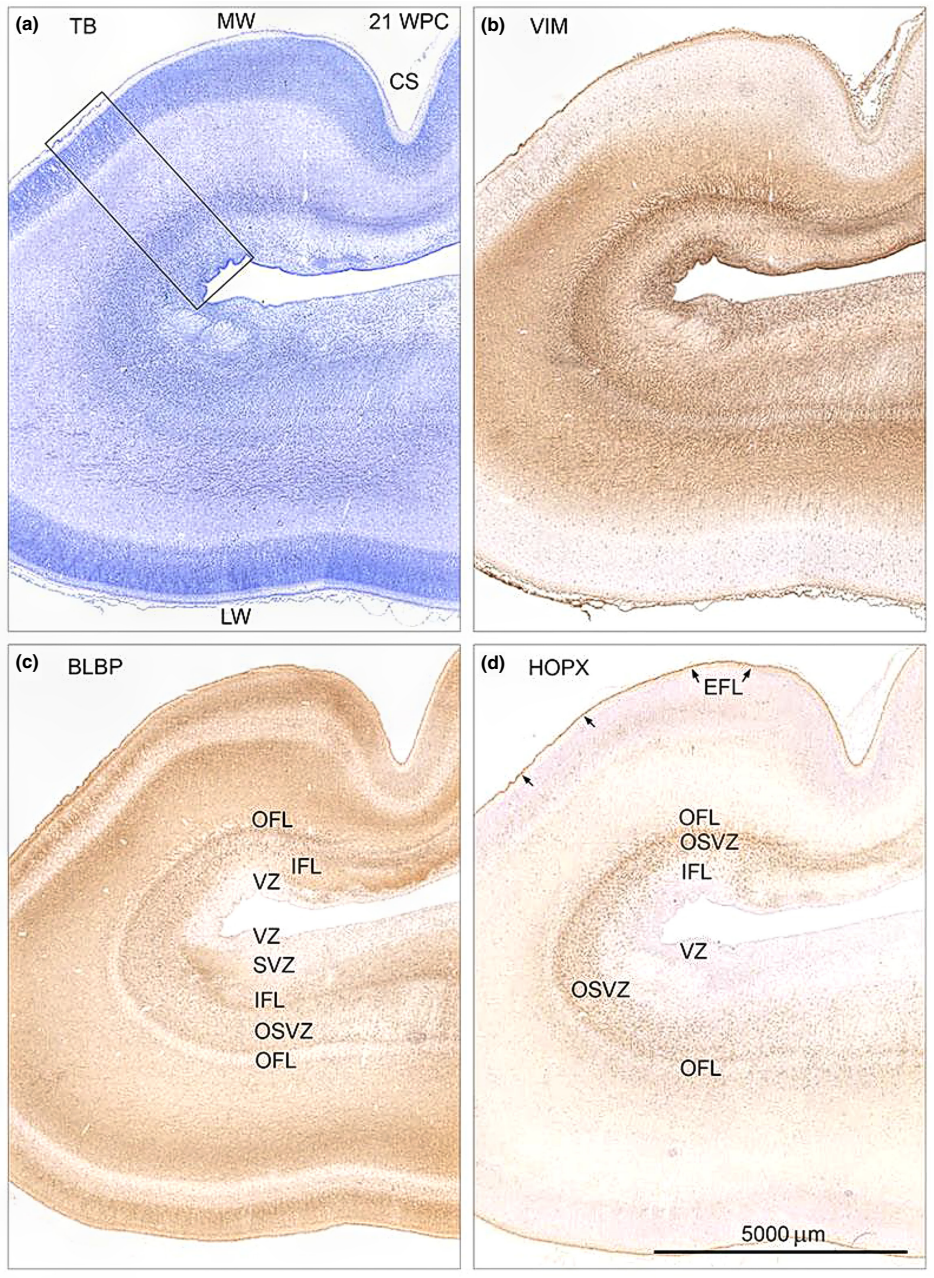
Distribution of toluidine blue (TB) (a), vimentin (VIM) (b), BLBP (c) and HOPX (d) immunoreactivity in consecutive coronal sections of occipital cortex from a 21 wpc fetus (CRL: 200 mm). HOPX, BLBP and vimentin are all present in the endfeet layer (EFL) (arrows in (d)) lining the pial surface and in a large proportion of cells in the outer subventricular zone (OSVZ), separating the inner‐ (IFL) and outer fibrous layer (OFL). The zonal configuration of OSVZ as depicted with VIM, BLBP and HOPX is attenuated towards the calcarine sulcus (CS) and a narrower possible OSVZ emerges below CS where the majority of HOPX‐positive cells is stained with less intensity and oriented tangentially as opposed to the radial orientation of HOPX‐positive cells in the adjacent OSVZ (not shown). The cortical plate as defined by TB is divided into two sections by BLBP. Vimentin stains the ventricular zone (VZ), which is almost devoid of HOPX and contains few BLBP‐positive cells. The framed area in (a) is shown in higher magnification in Figure 2. CS, calcarine sulcus; EFL, endfeet layer; IFL, inner fibrous layer; LW, lateral wall; MW, medial wall; OFL, outer fibrous layer; OSVZ, outer subventricular zone; SVZ, subventricular zone; TB, toluidine blue; VIM, vimentin; VZ, ventricular zone. All figures are of same magnification. Scale bar: 5000 μm.

**FIGURE 2:**
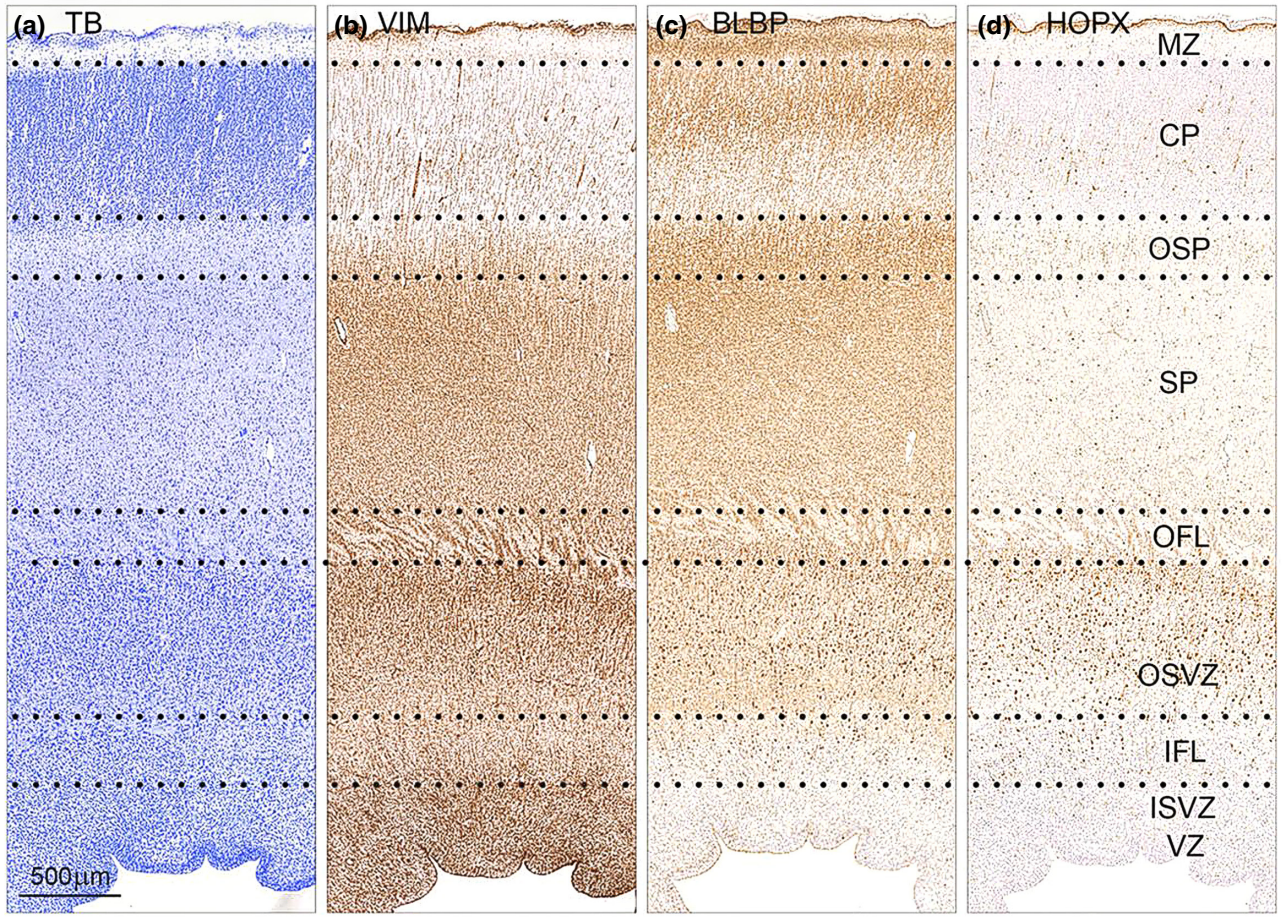
Distribution of toluidine blue (TB) (a), vimentin (b), BLBP (c) and HOPX (d) immunoreactivity in consecutive coronal sections of occipital cortex from a 21 wpc fetus (CRL: 200 mm). Described zones of the neocortex are depicted with dotted lines. At this magnification it is evident, that although staining of vimentin, BLBP and HOPX overlap, they have distinct patterns also described briefly in Figure 1. CP, cortical plate; IFL, inner fibrous layer; ISVZ, inner subventricular zone; MZ, marginal zone; OFL, outer fibrous layer; OSP, outer subplate; OSVZ, outer subventricular zone; SP, subplate; VZ, ventricular zone. All figures are of same magnification. Scale bar: 500 μm.

Given that much research on neocortical development and HOPX in fetal brain, has been performed on occipital and parietal cortex, we initially investigated HOPX as well as two canonical radial glial cell markers BLBP and vimentin, in the occipital lobe of the developing human brain at midgestation. Several overlapping terms describe the zones of the fetal neocortex (Table 1). The terminology put forth by Smart et al., 2002 (Smart et al., 2002) (Table 1; Figure 1c,d; Figure 2) is commonly used. Although their observations were made on monkey (Smart et al., 2002), definitions are similar to other recognized definitions (Bakken et al., 2016; Molnár et al., 2019) and comparable to observations in human fetal brain and were therefore selected to describe findings in this study.

**FIGURE 3:**
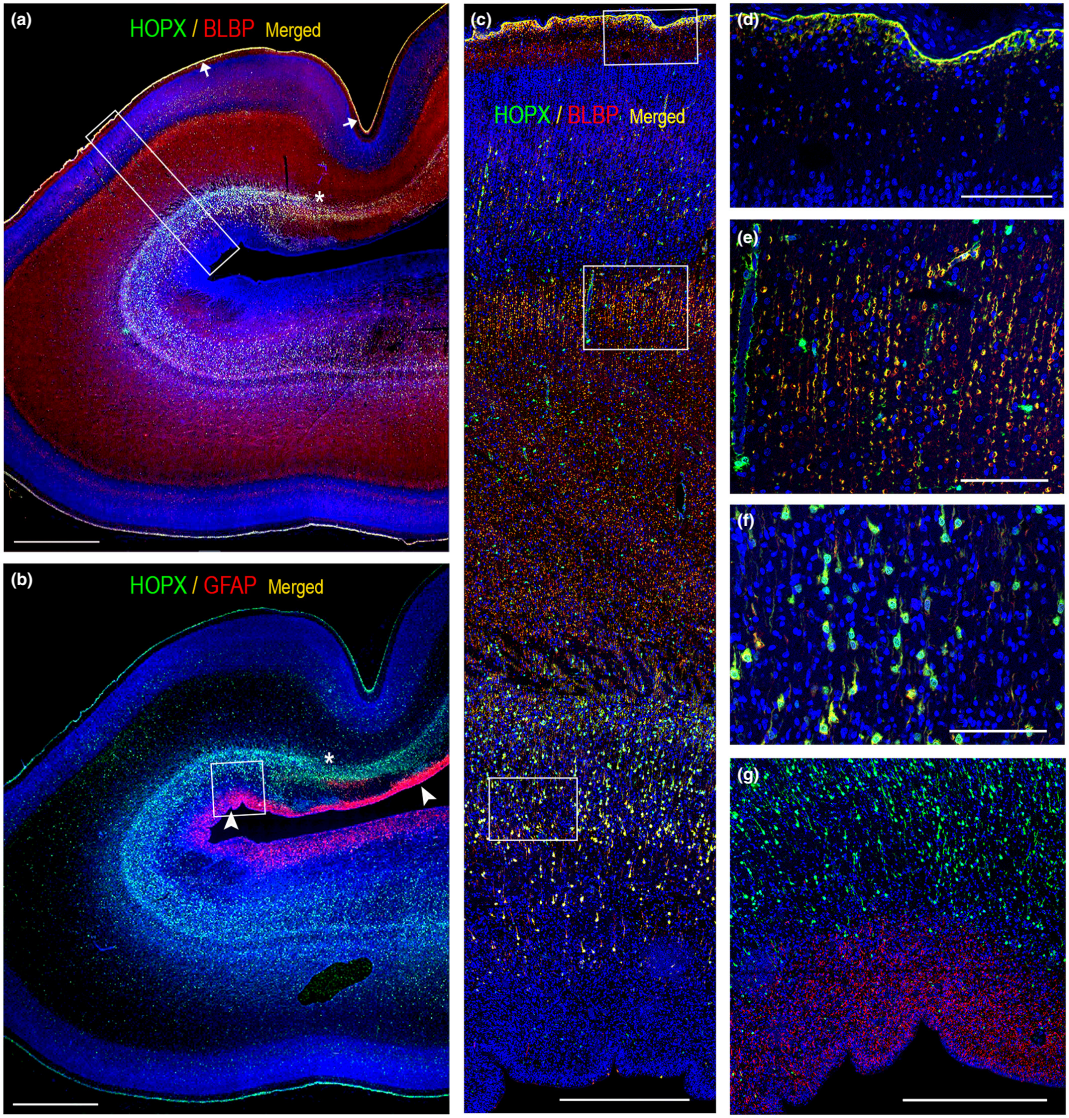
(a) and (b) are whole‐slide fluorescent scans of occipital cortex from the same 21 wpc fetus as shown in Figure 1. Sections are double‐labeled with antibodies against HOPX and BLBP (a) and HOPX and GFAP (b). In this particular section, GFAP reactive fibers are not seen at this low magnification. The asterisks in (a) and (b) mark a transition to a narrower outer subventricular zone below the calcarine sulcus. In (c), the boxed area in (a) is shown in higher magnification, revealing scattered HOPX‐positive cells and blood vessel walls in the subplate and cortical plate in addition to described findings in Figures 1 and 2. The HOPX and BLBP‐positive endfeet layer facing the subarachnoid space is depicted in (d) (upper boxed area in (c)); HOPX and BLBP‐positive fibers in the subplate in (e) (middle boxed area in (c)) and HOPX and BLBP‐positive radially oriented outer radial glial cells in (f) (similar area shown in lower boxed area in (c)). At this stage, HOPX is not present in the ISVZ and VZ as shown in (g) (boxed area in (b)), in contrast to GFAP, which is prevalent in both zones (arrowheads in b, g). Scale bars: (a, b) 2000 μm; (c, g) 500 μm; (d–f) 100 μm.

In the occipital lobe, HOPX, BLBP and vimentin immunoreactivity was present in a large proportion of cells in the outer subventricular zone (OSVZ) (Figure 1; Figure 2; Figure 3c,f), in radial glial fibers traversing the cortical wall (Figure 2; Figure 3c,e), as well as in the subpial radial glial endfeet layer (Figure 1; Figure 2; Figure 3a–d) facing the subarachnoid space. No ventricular radial connection was observed with HOPX‐staining and these observations corresponds to previous findings, establishing HOPX as a marker of outer radial glial cells (Pollen et al., 2015). Few HOPX‐ and BLBP‐positive cells were also scattered in the inner (IFL) and outer fibrous layer (OFL), subplate (SP) and cortical plate (CP) (Figure 1c,d; Figure 2c,d; Figure 3), although a proportion of these cells was only HOPX positive (data not shown). In accordance with previous findings (Nowakowski et al., 2016), HOPX immunoexpression was less prominent below the calcarine sulcus (CS) compared with the adjacent gyrus at midgestation (Figure 1d; Figure 2d; Figure 3a,b), supporting a role of HOPX‐positive oRGs in gyrification. A switch from radial to the mainly tangential orientation of HOPX+ cells (not shown) was also observed in this region, as well as a general narrowing of the SP and a condensed OSVZ (Figure 1; Figure 3a,b). Apart from radial glial cells, HOPX also stained blood vessel walls (Figure 3c,e).

Vimentin is an unspecific radial glial marker known to stain radial glial fibers and cells in the VZ (Stagaard & Møllgård, 1989), whereas HOPX has been shown to stain VZ in early corticogenesis (wpc 11.5) but fades towards midgestation (Pollen et al., 2015). Our findings (Figure 1b,d; Figure 2b,d; Figure 3a–c,g) were concordant with these observations and found BLBP to be absent in VZ at midgestation but present at earlier stages mimicking the pattern of HOPX (Figure 1c; Figure 2c; Figure 3a,c; not shown). The radial glial scaffold has been proposed to change around 17 gw (15 wpc) from a continuous scaffold with processes spanning the entire thickness of the telencephalic wall to a discontinuous structure with apical truncated radial glial cells terminating in the OSVZ (Nowakowski et al., 2016). In the same gestational period, Smart and colleagues described how the emerging IFL advances caudally extending over the occipital pole at E78 (~19 wpc) (Smart et al., 2002). These changes in zonal structure overlap with HOPX transition from VZ to OSVZ, and HOPX in early VZ may represent changes in radial glial cells moving them toward their fate as oRGs in OSVZ.

### 3.2 HOPX and neocortical uniformity

A presumption of cortical uniformity was commonly used in developmental neuroscience in the past. However, considering recently discovered interregional differences in cellular expression profiles and maturation status (Nowakowski et al., 2017) as well as described differences in zonal structure (Table 1) this view is now outdated.

**FIGURE 4:**
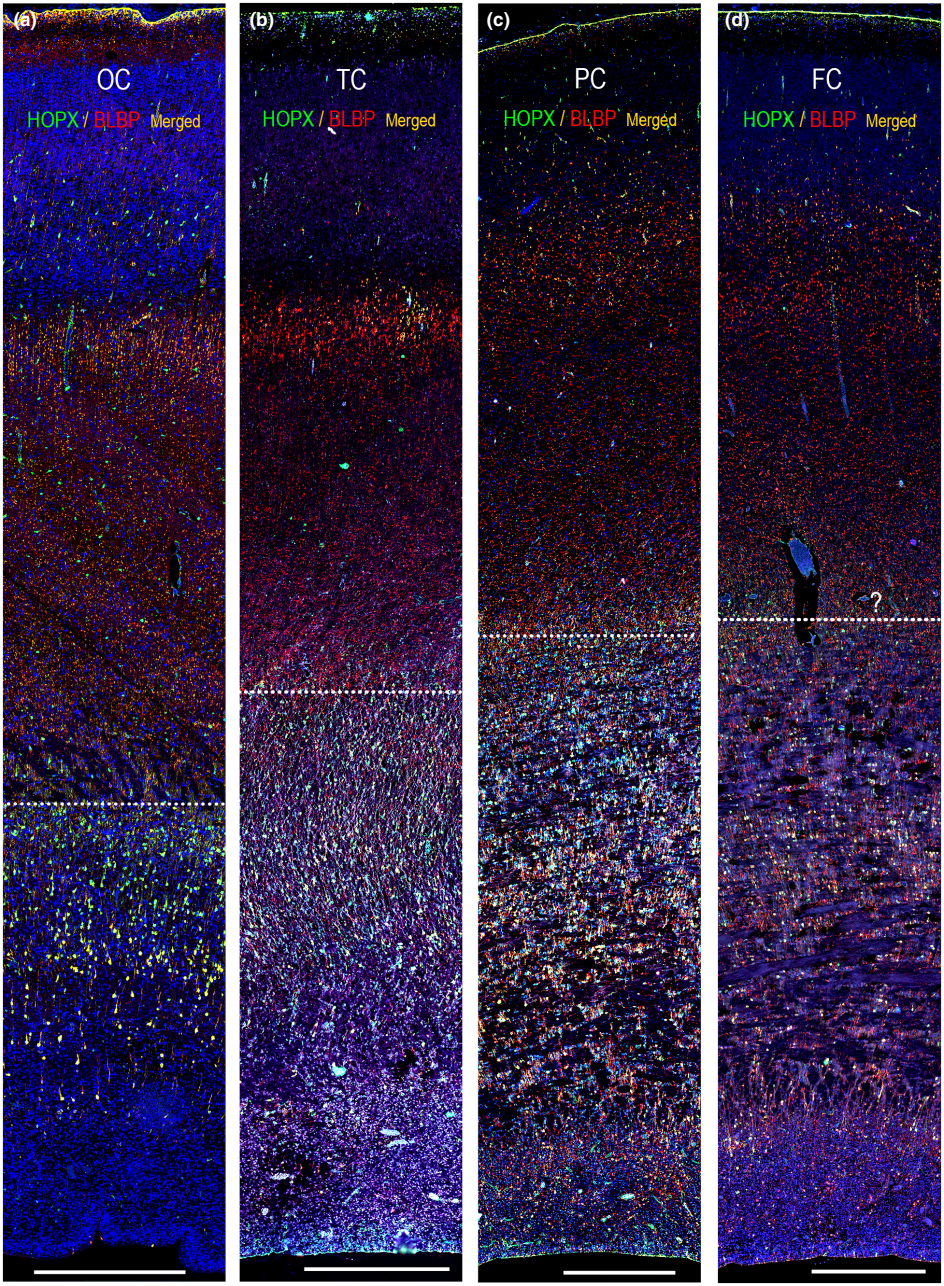
Immunofluorescent staining of HOPX and BLBP in coronal sections of occipital (a), temporal (b), parietal (c) and frontal (d) neocortex from 21 wpc (a) and 19 wpc (b–d) human fetal brains. The parietal cortex shown here has been sectioned close to the frontal lobe, which may explain their similar appearance. The outer fibrous layer (OFL) is visible in (a). The inner fibrous layer (IFL)/outer subventricular zone (OSVZ) seem to increase in size in parietal and frontal cortex with a higher density of horizontal fibers interdigitating with radially oriented radial glial cells and their extensions. The dotted line in (a–d) illustrates a proposed outer demarcation of the OSVZ. FC, frontal cortex; OC, occipital cortex; PC, parietal cortex; TC, temporal cortex. Scale bars: 500 μm.

Across midgestational frontal, parietal, temporal and occipital neocortex, HOPX and BLBP staining in the subpial endfeet layer, in radial glial fibers and outer radial glial cells in the outer subventricular zone (OSVZ), as well as HOPX distribution in blood vessel walls were similar (Figure 4). However, HOPX and BLBP were absent in ventricular (VZ) and inner subventricular zone (ISVZ) in occipital cortex (Figure 2c,d; Figure 3a–c,g; Figure 4), whereas HOPX immunoreactivity was seen in the ventricular lining and multiple blood vessels in ISVZ in temporal (Figure 4b) and parietal cortex (Figure 4c) and BLBP stained the ventricular lining and radial glial fibers in ISVZ (Figure 4b–d). HOPX stained cells were also less densely packed in OSVZ in frontal cortex compared with the other regions and the scattered HOPX‐positive cells and varicosities in OFL and SP were most evident in occipital cortex. Pollen and colleagues found HOPX to be most prevalent in OSVZ, and concordant with our results, HOPX was primarily seen in VZ in early corticogenesis (Pollen et al., 2015). The presence of HOPX in VZ of temporal and parietal cortex, may therefore represent regional differences in HOPX distribution, but could also reflect a maturation lag. Given that radial glial cells in frontal cortex exceed visual cortex radial glia maturation by approximately 3 weeks (Nowakowski et al., 2017) and occipital cortex shown in Figure 4 is 2 weeks older than frontal cortex (Figure 4a,d; 21 vs. 19 wpc), differences between these regions may not only reflect accelerated maturation in frontal cortex.

The total depth of the cerebral wall diminishes rostrocaudally in monkey (Smart et al., 2002). OSVZ is thicker round the occipital pole than in more rostral regions, whereas the CP and SP are thinner in caudal compared with more rostral regions (Smart et al., 2002) (Table 1). At E65 the OSVZ also has a higher cell packing density towards the pole of the occipital lobe (Smart et al., 2002). The described differences in laminar composition could therefore also explain what seems to be a lower density of HOPX in frontal lobe at midgestation (Figure 4).

Lateral to medial maturation gradients were not considered and differences in fiber connections, function and cytoarchitecture of cortical areas within and between the interrogated regions may also influence results, making it difficult to extrapolate findings to represent general differences between cortical lobes.

**FIGURE 5:**
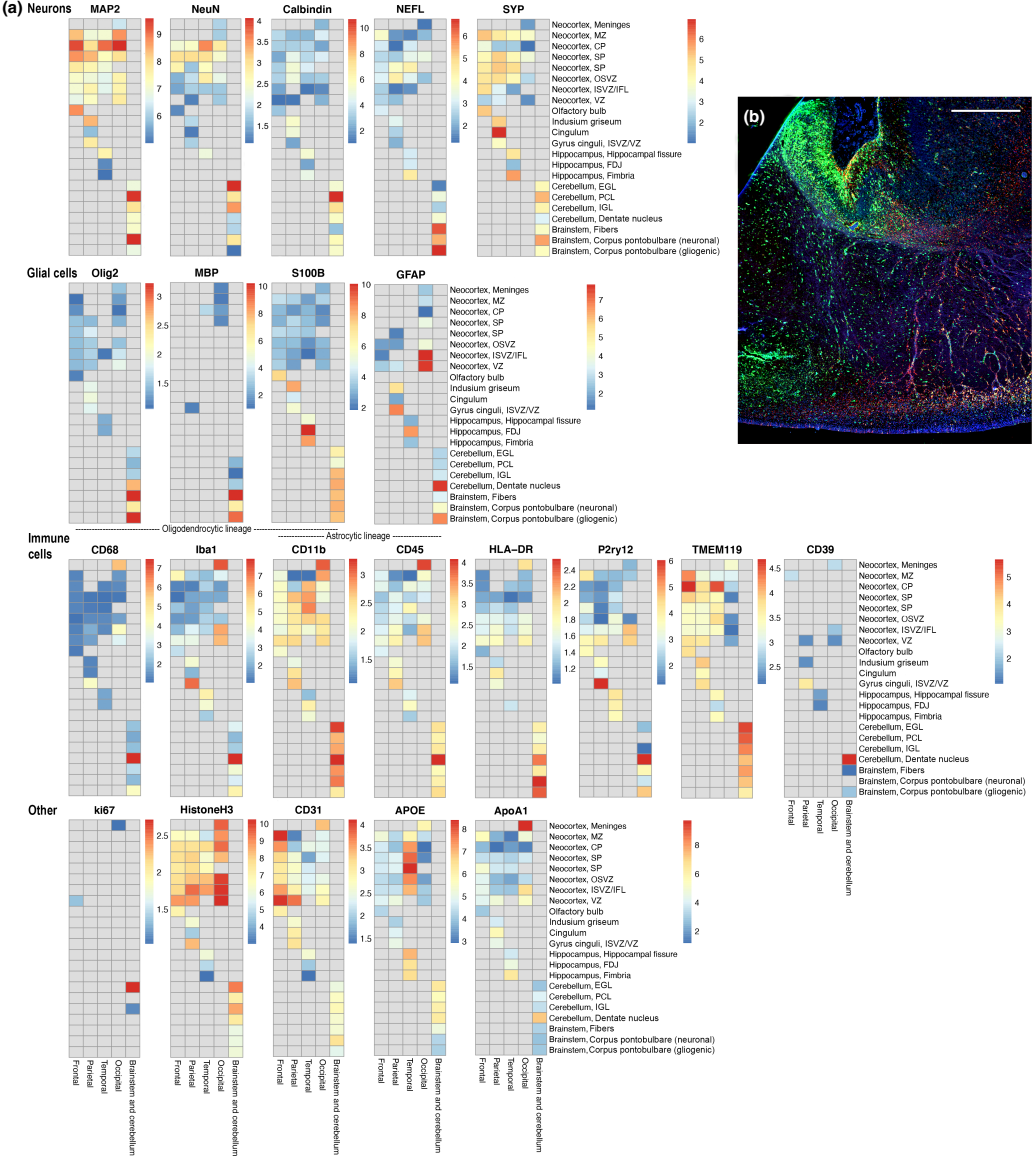
(a) Each heatmap shows log2 signal‐to‐noise ratios of different proteins in 22 regions of interest (ROI) (rows) in selected areas/regions (columns) from 13 wpc (frontal), 19 wpc (temporal, parietal) and 21 wpc (occipital, brain stem and cerebellum) human fetal brain samples profiled using Nanostring GeoMx^**®**^. Values below 1 or missing are depicted in grey. Proteins are grouped according to cell types in which they are known to be prevalent. Apart from indusium griseum (b) (green, HOPX; red, BLBP), examples of selected areas/regions are illustrated on Figure 1, 2 (neocortical zones), Figure 6 (olfactory bulb), Figure 7 (hippocampus), Figure 8 (cerebellar cortex, center of dentate nucleus, axonal fibers and corpus pontobulbare). All regions of interest are depicted in Figure S1–S5. The screening reveals differences in protein abundance between regions/areas and/or ages in fetal developing brain. Scale bar: 500 μm.

To further interrogate general spatiotemporal differences in cell/protein composition in developing human neocortex, we screened a fixed panel of proteins in different zones of frontal (13 wpc), parietal (19 wpc), temporal (19 wpc) and occipital (21 wpc) cortical wall as well as in the brain stem and cerebellum (Figure 5) using Nanostring GeoMx® Digital Spatial Profiling (DSP). The most evident differences in protein distribution in neocortex were higher immunoexpression of GFAP in VZ and ISVZ of occipital cortex compared with the three other regions (Figure 5); a higher immunoexpression of CD31 (endothelial cells marker) in frontal cortex; a general lower immunoexpression of TMEM119 (microglial marker) in occipital cortex and higher immunoexpression of APOE (apolipoprotein) in temporal cortex (Figure 5).

GFAP, an intermediate filament protein, has been used as a marker for radial glial cells, transitional forms and different types of astrocytes (Holst et al., 2019). Comparing DSP data (Figure 5) and immunofluorescence (Figure 3b,g), GFAP protein was abundant in VZ and ISVZ in occipital cortex, compared with other zones or regions of neocortex (Figure 3b, Figure 5, Figure 7a) (note, that VZ of temporal cortex and IZ of occipital cortex were not evaluated with DSP), and it did not seem to overlap with HOPX immunoexpression in occipital lobe at midgestation (Figure 3b,g), indicating that GFAP may preferentially stain classical or/and apical truncated radial glial cells in occipital VZ and ISVZ and that GFAP is not highly immunoexpressed in outer radial glial cells in neocortex at the investigated developmental stages and regions. However, regional differences in GFAP staining may also partly reflect temporal changes in GFAP expression, as indicated by previous results depicting GFAP immunoreactivity in VZ at 12 wpc (Holst et al., 2019). Recent findings of laminar and regional heterogeneity of astrocytes in mammalian cerebral cortex are suggested to be partly mediated by neuronal cues (Bayraktar et al., 2020). However, it is also plausible that early regional patterning in heterogeneous radial glial cells contributes to complex astrocyte diversity, emphasizing the importance of regional specification in developmental neuroscience.

Hypothesized regional and/or temporal differences in glial composition, vessel density, immune cell diversity and presence of apolipoproteins (Figure 5) determined by DSP are preliminary. Since the measured protein differences are relative, we cannot discriminate general low expression and therefore possibly insignificant differences from clinically relevant findings.

### 3.3 HOPX in the limbic system

**FIGURE 6:**
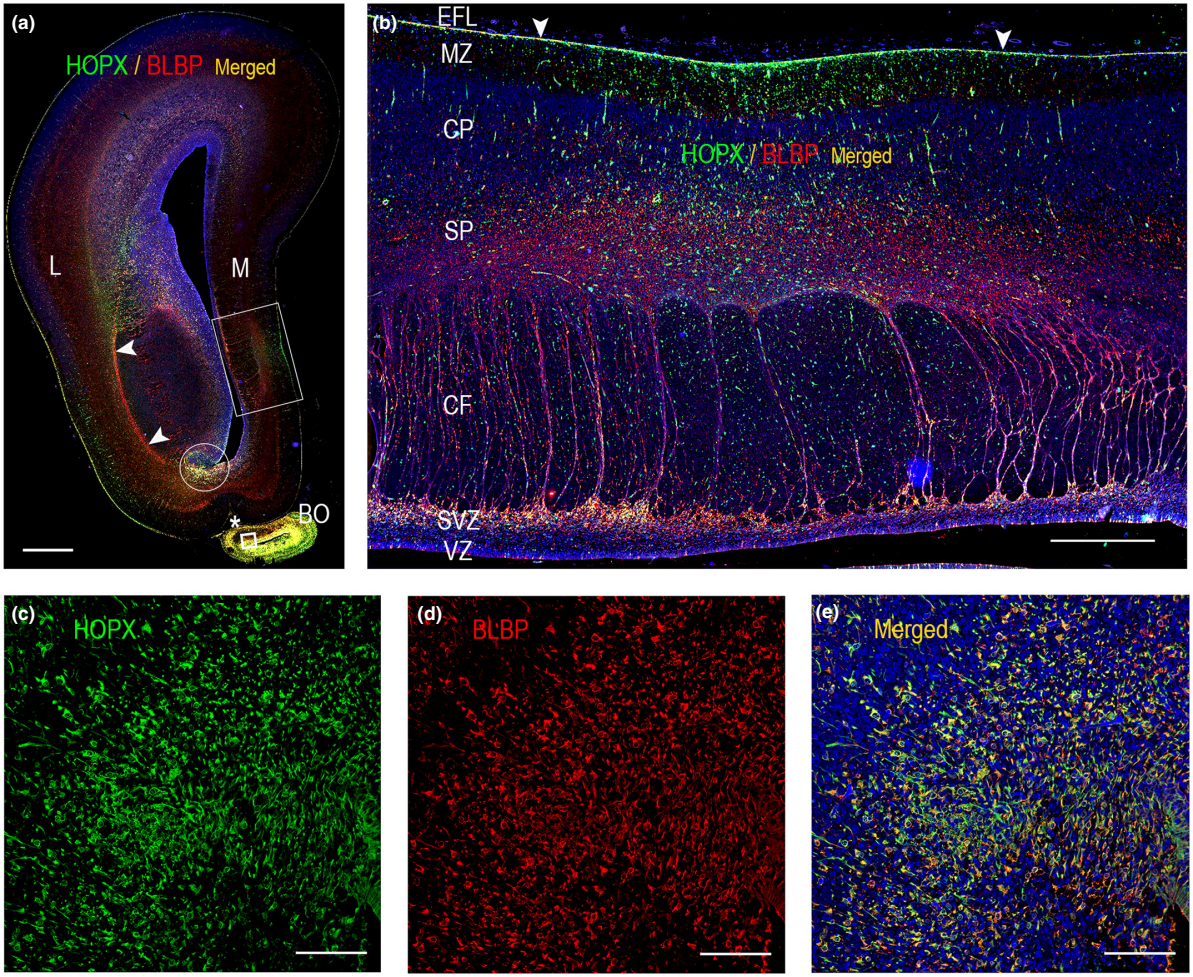
Whole‐slide fluorescent scans (a, b) and higher magnifications (c, d, e) of HOPX and BLBP in frontal cortex from a 19 wpc human fetus (a). Both HOPX and BLBP are prevalent in the olfactory bulb (lower boxed area in (a) shown in higher magnification in c, d, e) and in relation to the rostral extension of the caudal ganglionic eminence (circle in (a)), whereas the signal is sparse in lateral ganglionic eminence. BLBP immunostaining is prominent in the radial glial fiber fascicle (arrowheads) and HOPX delineates the pial border through an immunoreactive endfeet layer, which is accentuated in “limbic cortex” (between arrowheads in higher magnification in (b)). In this region, HOPX immunoreactivity is also scattered throughout the cortical wall. BO, olfactory bulb; CF, callosal fibers; CP, cortical plate; EFL, endfeet layer; L, lateral; M, medial; MZ, marginal zone; SP, subplate; SVZ, subventricular zone; VZ, ventricular zone. Scale bars: (a) 2000 μm; (b) 500 μm; (c), (d, e) 100 μm.

**FIGURE 7:**
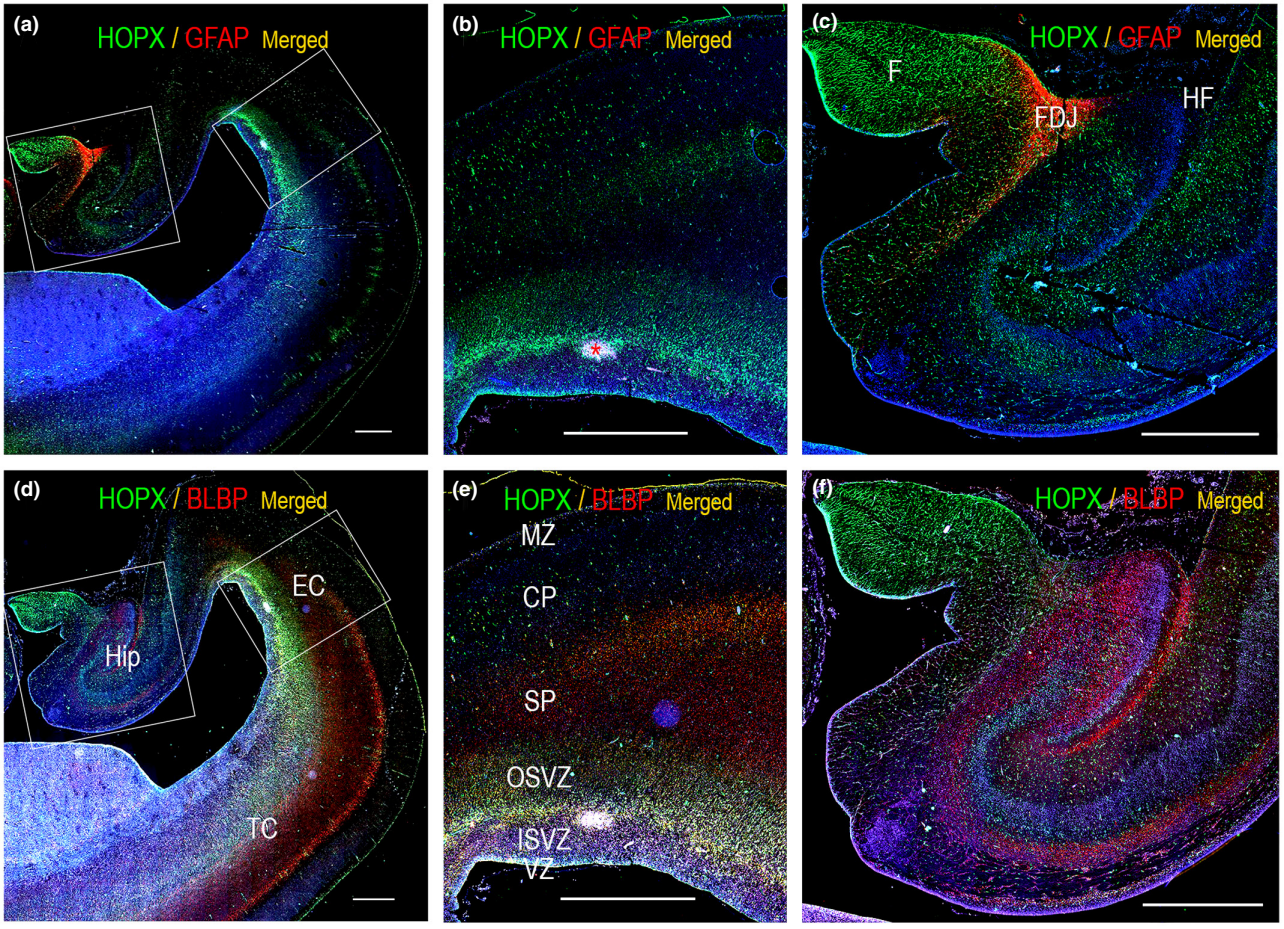
(a) and (d) are whole‐slide fluorescent scans of the hippocampal formation and temporal cortex from a 19 wpc human fetus. Sections are double labelled with antibodies against HOPX and GFAP (a) and HOPX and BLBP (d). In (b and c), the boxed areas in (a) are depicted. (e) and (f) are higher magnifications of the boxed areas in (d). HOPX immunoreactivity is strongest in fimbria (F) (c, f), in subventricular zone (SVZ) in entorhinal cortex (EC) (b, e) and in the glial endfeet layer facing the subarachnoid space (a, d). GFAP is practically confined to the fimbrio‐dentate junction (FDJ) (a, c). BLBP and HOPX overlap in SVZ, whereas they seem to stain different cell populations in the hippocampus. Of note, the asterisk in (b) indicates presence of intraventricular hemorrhage. CP, cortical plate; EC: entorhinal cortex; F: fimbria; FDJ: fimbrio‐dentate junction; HF: hippocampal fissure; Hip, hippocampus; ISVZ, inner subventricular zone; MZ, marginal zone; OSVZ, outer subventricular zone; SP, subplate; TC, temporal cortex; VZ, ventricular zone. Scale bars: 1000 μm.

In other cortical regions, lamination may be even more complex than in neocortex and the distribution of radial glial cells less extensively studied. To define HOPX landscape in cortical regions diverging from neocortical organization, we interrogated HOPX and BLBP immunoreactivity in limbic structures, including limbic cortex in the frontal lobe (Figure 6b), olfactory bulb (Figure 6c–e), entorhinal cortex (Figure 7e), hippocampus (Figure 7f) and indusium griseum (Figure 5). Of note, olfactory bulb is a complex structure, and may not be perceived as true cortex. In whole‐slide immunofluorescent scans of frontal (Figure 6a) and temporal (Figure 7a) lobes, these regions of the limbic system displayed strong HOPX immunoreactivity, more prominent than in adjacent neocortex (Figure 6, Figure 7, not shown). BLBP and HOPX immunoexpression were similar in most regions but did not completely overlap.

In frontal limbic cortex, HOPX stained cells throughout the cerebral wall, except from VZ and the inner part of the SVZ (Figure 6b). HOPX was accentuated towards the pial border, but whereas intracerebral vessels were HOPX positive, blood vessels in the subarachnoid space did not show HOPX immunoreactivity (Figure 6b).

In developing human olfactory bulb, HOPX and BLBP staining were prominent and overlapped (Figure 6a,c–e), with strongest expression in the central core, suggesting that these cells represent radial glial cells at midgestation. HOPX has been found to be expressed by astrocytes, but not neurons in mouse olfactory bulbs (Li et al., 2015).

According to Bobić Rasonja et al. (2019), the human indusium griseum (IG) is the ventromedial continuation of the cingulate gyrus covering the dorsal surface of the corpus callosum (Bobić Rasonja et al., 2019). This may be debated, but in any case, IG is considered a dorso‐medial rudimentary part of the hippocampus. We found HOPX in all three transient laminae of the developing human IG, as well as in scattered cells and blood vessel walls in the underlying corpus callosum at midgestation (Figure 5), a time of differentiation and reorganization in IG (Bobić Rasonja et al., 2019). DSP revealed some extent of immunoexpression of MAP2, synaptophysin and GFAP in IG − all of which have been found in developing IG by Rasonja and colleagues (Bobić Rasonja et al., 2019). BLBP mainly stained part of the IG MZ, as well as the SP and SVZ in the adjacent cingulate gyrus, where HOPX staining was less prominent (Figure 5).

In the hippocampus, BLBP and HOPX stained different cell populations. HOPX immunoexpression was most prominent in fimbria. Initially, the developing fimbria is a neuron‐free gliogenic region (Stagaard Janas et al., 1991). DSP showed lack of neuronal markers MAP2 and NeuN and oligodendrocyte markers Olig2 and MBP in fimbria (Figure 5), as well as low immunoexpression of GFAP (Figure 5), which was confirmed with immunofluorescence (Figure 7a,c, Figure S3). GFAP was however present in developing fimbrial VZ (Figure S3). S100 is a known marker of radial glial cells, astrocytes, and oligodendrocytes (Holst et al., 2019) and was seen in both fimbria and the fimbriodentate junction in DSP analysis (Figure 5), as found previously in the same material (Holst et al., 2019). In developing hippocampus, HOPX+ progenitors have been suggested to give rise to both neurons and glial cells and to migrate from the ammonic neuroepithelium towards the future dentate gyrus, with the majority of HOPX+ progenitors placed in the cornu ammonis (CA) at 20 wpc (Zhong et al., 2020). In concordance with these findings, we found both HOPX‐positive and BLBP‐positive cells in CA at midgestation (Figure 7d,f), although they seemed to prefer different layers and generally spared the future pyramidal cell layer in CA2 and CA1 (Figure 7c,f). In the adult mouse hippocampus, HOPX is seen in mature astrocytes, but not in oligodendrocytes or neurons; HOPX+ astrocytes are mostly located in CA regions and strongly expressed in the subgranular zone of the dentate gyrus, a region of adult neurogenesis (Li et al., 2015).

In entorhinal cortex, HOPX and BLBP both stained the VZ and SVZ, radial glial fibers and the subpial endfeet layer (Figure 7b,e), very similar to the distribution in neocortex, albeit with a stronger expression than adjacent temporal neocortex (Figure 7a,d).

Collectively, our results support HOPX as a marker of radial glial cells and the astroglial lineage in cortical regions. However, HOPX did not overlap completely with GFAP immunostaining in the fimbriodentate junction, which also displays high S100 immunoexpression (Figure 5, (Holst et al., 2019)), emphasizing that HOPX only stains fractions of astrocytes. HOPX also diverged from BLBP staining in some areas, indicating that HOPX marks subgroups of radial glial cells or more differentiated progeny. Of note, radial glial cells also give rise to Tbr2 expressing intermediate progenitor cells in SVZ contributing substantially to neurogenesis (Vasistha et al., 2015). Their relation to HOPX was not interrogated.

### 3.4 HOPX in cerebellum and brain stem

Further interrogating the hypothesis of HOPX expression in radial glial cells in regions of the brain outside neocortex, we looked to cerebellar cortex. Bergmann glial cells (BG) are cerebellar radial glial cells (Barry et al., 2014). Cerebellar classical radial glial cells generate BGs through loss of their apical processes and somal relocation to a basal position (to the prospective Purkinje cell (PC) layer) (Heng et al., 2017). BGs therefore resemble outer radial glial cells in human developing neocortex. Both oRGs and BGs have been implicated in folding of cortex (Borrell & Götz, 2014; Heng et al., 2017; Rash et al., 2019) and mouse BGs and human oRGs share molecular characteristics (Heng et al., 2017). BLBP is a well‐known BG marker present in BG fibers, endfeet, cell nuclei and cytoplasm (Feng et al., 1994). In developing mouse cerebellum, HOPX, BLBP and Sox2 have been found to co‐localize in presumptive BGs and HOPX and BLBP expression to be preserved in both nascent and mature BGs (Heng et al., 2017). In adult mice, findings suggest HOPX be expressed in the cerebellum in both PCs and BGs (Li et al., 2015).

**FIGURE 8:**
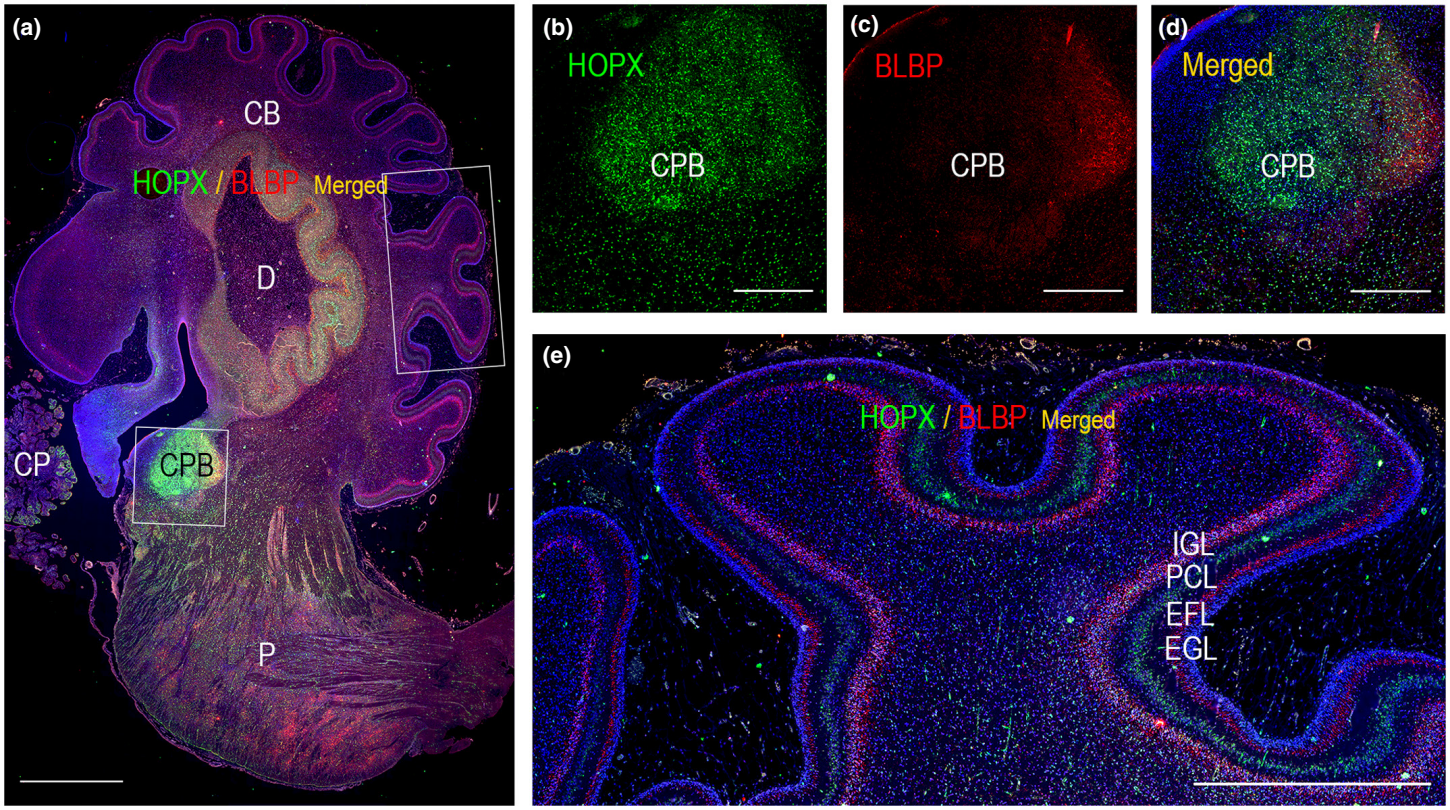
Distribution of HOPX and BLBP in cerebellum and brain stem from a 21 wpc human fetal brain. (b–e) are higher magnifications of boxed areas in (a). HOPX is most prevalent in corpus pontobulbare (CPB) depicted in (a–d**)**. HOPX (b) and BLBP (c) seem to occupy different regions (d) of this cellular aggregate dominated by strong HOPX immunoreactivity. HOPX is also scattered in the brain stem and in the dentate nucleus (a) as well as in blood vessel walls (e). In the cerebellar cortex HOPX is mostly confined to the Purkinje cell layer (e). CB, cerebellum; CP, choroid plexus; CPB, corpus pontobulbare; D, dentate nucleus; EFL, endfeet layer; EGL: external granular/germinal layer; IGL: internal granular layer; P, pons; PCL: Purkinje cell layer. Scale bars: (a): 2000 μm; (b–e) 1000 μm.

Although we cannot exclude weak HOPX staining in human BGs at midgestation, we mainly found HOPX immunoexpression in the PC layer (PCL) and in blood vessel walls in the human cerebellum at this timepoint (Figure 8a,e). HOPX was also found to stain part of the dentate nucleus (Figure 8a). As expected, BLBP immunoreactivity was mainly seen below the PC layer in the granular cell layer as well as in fibers traversing the molecular zone towards the transitional external granular layer/external germinal layer, indicative of BGs and their radial fibers. Discrepancies between mice and human samples may be a matter of differences in HOPX expression between species or temporal changes in BG HOPX immunoexpression.

In the brain stem, HOPX was highly immunoexpressed in corpus pontobulbare (Figure 8b,d) but also scattered throughout the brain stem (Figure 8a). Corpus pontobulbare, a transient fetal structure derived from the lower rhombic lip (Zec et al., 1997), is described as a fetal proliferative zone (Yachnis & Rorke, 1999) comprising immature premigrational neurons and glia (Zec et al., 1997). HOPX and BLBP seemed to occupy different regions of this cellular aggregate (Figure 8b–d) dominated by strong HOPX immunoreactivity. GFAP was most prominent in a third region (Figure S5). Cells migrate from corpus pontobulbare between 8–20 weeks of gestation to form the inferior olivary nuclei, arcuate nuclei and basal pontine nuclei (Yachnis & Rorke, 1999). At midgestation, HOPX‐positive cells were also scattered throughout the brain stem, particularly below corpus pontobulbare. We speculate that these cells may be migratory cells originating in the corpus pontobulbare, although this hypothesis requires further investigation. DSP data from two different regions of corpus pontobulbare reveal higher MAP2, NeuN and Synatophysin and lower Olig2, MBP and GFAP immunoexpression (Figure 5) in the region with prominent HOPX immunoexpression compared with the region with high GFAP immunoexpression (Figure S5), indicating regional differences in cellular composition in corpus pontobulbare.

### 3.5 HOPX in brain barriers

**FIGURE 9:**
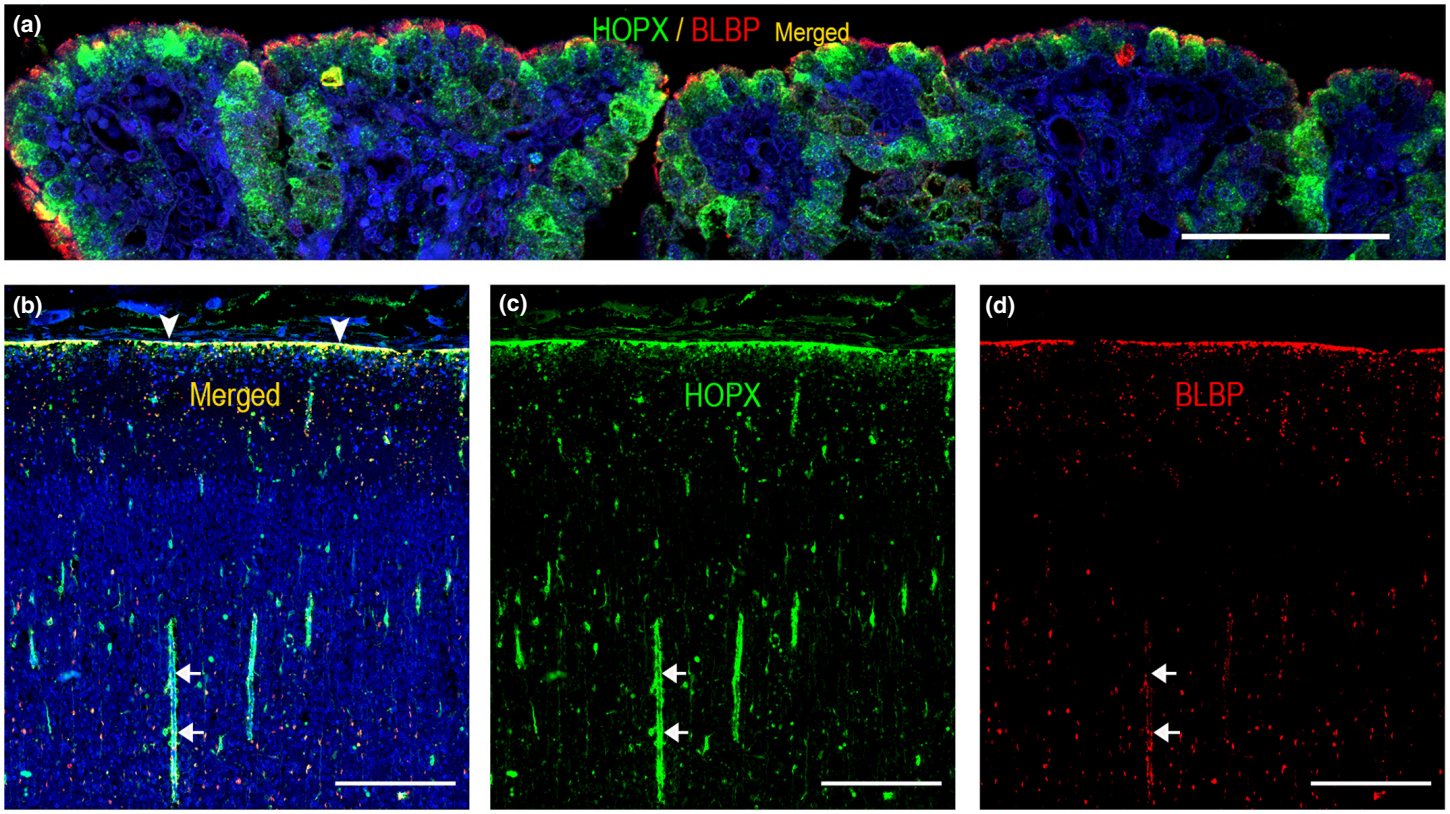
HOPX and BLBP immunoreactivity in brain barrier interfaces in a 21 wpc (a) and a 19 wpc (b–d) human fetal brain. A granular cytoplasmatic stain defines HOPX immunoreactivity in the majority of choroid plexus epithelial cells (a) (blood‐CSF interface at the fourth ventricle). BLBP on the other hand only stains the apical membrane of the choroid plexus epithelial cells, which is also stained by HOPX. The stroma including vasculature is unstained for both HOPX and BLBP. In (b, c) HOPX is seen in the glial endfeet layer facing the subarachnoid space (SAS, arrowheads) (outer CSF‐brain barrier) as well as in epithelial cells of penetrating arterioles (arrows) and multiple capillaries (blood–brain barrier proper) in the cortical wall. Note the HOPX immunoreactivity of leptomeningeal cells in the SAS and the absence of BLBP staining. BLBP is equally present in the endfeet layer and in endfeet surrounding vasculature, but not in the vasculature wall per se (arrows). (d). Scale bars: (a) 100 μm; (b–d): 200 μm.

Investigating alternative functions of HOPX in developing human brain, we found HOPX in brain barrier interfaces. HOPX and BLBP immunoreactivity was seen in most choroid plexus epithelial cells (Figure 9a) (blood‐CSF interface at the fourth ventricle), in the glial endfeet layer facing the subarachnoid space (outer CSF‐brain barrier) (Figure 9b–d) and HOPX was also present in endothelial cells of penetrating arterioles and multiple capillaries (blood–brain barrier proper) (Figure 9c). Claudins are a large family of membrane proteins regulating the permeability of tight junctions (Günzel & Yu, 2013) and important for brain barrier function (Günzel & Yu, 2013; Scalise et al., 2021). HOPX has previously been found to regulate claudin expression in intestinal epithelial cells (Lili et al., 2016) but whether HOPX also regulates claudin expression in developing brain barriers requires further investigation.

### 3.6 HOPX in human embryonic and early fetal development

Scrutinizing HOPX immunoexpression in human embryos and early fetuses, HOPX was seen in the brain by the end of the embryonic stage in the VZ of the lower brain stem and subsequently in spinal cord. In early fetuses, this was followed by staining of choroid plexus epithelial cells (initially in the 4th ventricle), endothelial cells, VZ and later SVZ in neocortex, antiHem, olfactory bulb and migrating cells in brain stem and cerebellum. Interestingly, we also found strong HOPX immunoreaction in the dorsal aorta corresponding to the AGM (aorta‐gonad‐mesonephros) stem cell system at 5 wpc, prior to HOPX presence in CNS. The AGM region generates hematopoietic stem cells migrating to the fetal liver (Gao et al., 2018). HOPX has recently been implicated in both normal and malignant hematopoiesis and is suggested to impact hematopoietic stem cell stemness and quiescence (Lin et al., 2020), which could explain HOPX presence in the AGM system.

## 4 CONCLUSION AND PERSPECTIVES

Our findings show HOPX presence in outer radial glial cells in several human developing brain regions as well as in cells in gliogenic regions but also paint a more complex picture of HOPX in developing brains, staining a range of cell types and specific regions. In different organs and tissues (heart, lung, lymphatic tissue, intestine and brain), HOPX has been proposed to be involved in cell proliferation, differentiation, maturation, quiescence, apoptosis and more (Liu & Zhang, 2020). This evidence portrays HOPX as a pleiotropic protein, which due to its specific presence in key areas of the CNS, could have implications for the development and progression of neurological and psychiatric disorders.

Performing Nanostring GeoMx^®^ Digital Spatial Profiling using biobanked human fetal sections was feasible and therefore opens new avenues for the use of this invaluable material.

## Supporting information


Figure S1
Figure S2
Figure S3
Figure S4
Figure S5
Table S1

## Data Availability

The data that support the findings of this study are available from the corresponding author upon reasonable request.

## References

[joa13844-bib-0001] Altman, J. & Bayer, S.A. (2002) Regional differences in the stratified transitional field and the honeycomb matrix of the developing human cerebral cortex. Journal of Neurocytology, 31, 613–632. 14501203 10.1023/a:1025787427576

[joa13844-bib-0002] Bakken, T.E. , Miller, J.A. , Ding, S.L. , Sunkin, S.M. , Smith, K.A. , Ng, L. et al. (2016) A comprehensive transcriptional map of primate brain development. Nature, 535, 367–375. 27409810 10.1038/nature18637 PMC5325728

[joa13844-bib-0003] Barry, D.S. , Pakan, J.M. & Mcdermott, K.W. (2014) Radial glial cells: key organisers in CNS development. The International Journal of Biochemistry & Cell Biology, 46, 76–79. 24269781 10.1016/j.biocel.2013.11.013

[joa13844-bib-0004] Bayraktar, O.A. , Bartels, T. , Holmqvist, S. , Kleshchevnikov, V. , Martirosyan, A. , Polioudakis, D. et al. (2020) Astrocyte layers in the mammalian cerebral cortex revealed by a single‐cell in situ transcriptomic map. Nature Neuroscience, 23, 500–509. 32203496 10.1038/s41593-020-0602-1 PMC7116562

[joa13844-bib-0005] Bhaduri, A. , Sandoval‐Espinosa, C. , Otero‐Garcia, M. , Oh, I. , Yin, R. , Eze, U.C. et al. (2021) An atlas of cortical arealization identifies dynamic molecular signatures. Nature, 598, 200–204. 34616070 10.1038/s41586-021-03910-8 PMC8494648

[joa13844-bib-0006] Bobić Rasonja, M. , Orešković, D. , Knezović, V. , Pogledić, I. , Pupačić, D. , Vukšić, M. et al. (2019) Histological and MRI study of the development of the human indusium griseum. Cerebral Cortex, 29, 4709–4724. 30722016 10.1093/cercor/bhz004

[joa13844-bib-0007] Borrell, V. & Götz, M. (2014) Role of radial glial cells in cerebral cortex folding. Current Opinion in Neurobiology, 27, 39–46. 24632307 10.1016/j.conb.2014.02.007

[joa13844-bib-0008] Bystron, I. , Blakemore, C. & Rakic, P. (2008) Development of the human cerebral cortex: Boulder Committee revisited. Nature Reviews Neuroscience, 9, 110–122. 18209730 10.1038/nrn2252

[joa13844-bib-0009] Couturier, C.P. , Ayyadhury, S. , Le, P.U. , Nadaf, J. , Monlong, J. , Riva, G. et al. (2020) Single‐cell RNA‐seq reveals that glioblastoma recapitulates a normal neurodevelopmental hierarchy. Nature Communications, 11, 3406. 10.1038/s41467-020-17186-5 PMC7343844 32641768

[joa13844-bib-0010] Falcone, C. , Penna, E. , Hong, T. , Tarantal, A.F. , Hof, P.R. , Hopkins, W.D. et al. (2021) Cortical interlaminar astrocytes are generated prenatally, mature postnatally, and express unique markers in human and nonhuman primates. Cereb Cortex, 31, 379–395. 32930323 10.1093/cercor/bhaa231 PMC7947181

[joa13844-bib-0011] Feng, L. , Hatten, M.E. & Heintz, N. (1994) Brain lipid‐binding protein (BLBP): a novel signaling system in the developing mammalian CNS. Neuron, 12, 895–908. 8161459 10.1016/0896-6273(94)90341-7

[joa13844-bib-0012] Gao, X. , Xu, C. , Asada, N. & Frenette, P.S. (2018) The hematopoietic stem cell niche: from embryo to adult. Development, 145, dev139691. 29358215 10.1242/dev.139691 PMC5825844

[joa13844-bib-0013] García‐Moreno, F. , Vasistha, N.A. , Trevia, N. , Bourne, J.A. & Molnár, Z. (2012) Compartmentalization of cerebral cortical germinal zones in a lissencephalic primate and gyrencephalic rodent. Cerebral Cortex, 22, 482–492. 22114081 10.1093/cercor/bhr312

[joa13844-bib-0014] Günzel, D. & Yu, A.S. (2013) Claudins and the modulation of tight junction permeability. Physiological Reviews, 93, 525–569. 23589827 10.1152/physrev.00019.2012 PMC3768107

[joa13844-bib-0015] Heng, X. , Guo, Q. , Leung, A.W. & Li, J.Y. (2017) Analogous mechanism regulating formation of neocortical basal radial glia and cerebellar Bergmann glia. eLife, 6, 1–30. 10.7554/eLife.23253 PMC5457141 28489004

[joa13844-bib-0016] Holst, C.B. , Brøchner, C.B. , Vitting‐Seerup, K. & Møllgård, K. (2019) Astrogliogenesis in human fetal brain: complex spatiotemporal immunoreactivity patterns of Gfap, S100, AQP4 and YKL‐40. Journal of Anatomy, 235, 590–615. 30901080 10.1111/joa.12948 PMC6704246

[joa13844-bib-0017] Kelava, I. , Reillo, I. , Murayama, A.Y. , Kalinka, A.T. , Stenzel, D. , Tomancak, P. et al. (2012) Abundant occurrence of basal radial glia in the subventricular zone of embryonic neocortex of a lissencephalic primate, the common marmoset Callithrix jacchus. Cerebral Cortex, 22, 469–481. 22114084 10.1093/cercor/bhr301 PMC3256412

[joa13844-bib-0018] Kim, H.J. , Park, J.W. & Lee, J.H. (2020) Genetic architectures and cell‐of‐origin in glioblastoma. Frontiers in Oncology, 10, 615400. 33552990 10.3389/fonc.2020.615400 PMC7859479

[joa13844-bib-0019] Kostović, I. , Išasegi, I. & Krsnik, Ž. (2019) Sublaminar organization of the human subplate: developmental changes in the distribution of neurons, glia, growing axons and extracellular matrix. Journal of Anatomy, 235, 481–506. 30549027 10.1111/joa.12920 PMC6704274

[joa13844-bib-0020] Kostović, I. , Judas, M. , Rados, M. & Hrabac, P. (2002) Laminar organization of the human fetal cerebrum revealed by histochemical markers and magnetic resonance imaging. Cerebral Cortex, 12, 536–544. 11950771 10.1093/cercor/12.5.536

[joa13844-bib-0021] Li, D. , Takeda, N. , Jain, R. , Manderfield, L.J. , Liu, F. , Li, L. et al. (2015) Hopx distinguishes hippocampal from lateral ventricle neural stem cells. Stem Cell Research, 15, 522–529. 26451648 10.1016/j.scr.2015.09.015 PMC4704104

[joa13844-bib-0022] Lili, L.N. , Farkas, A.E. , Gerner‐Smidt, C. , Overgaard, C.E. , Moreno, C.S. , Parkos, C.A. et al. (2016) Claudin‐based barrier differentiation in the colonic epithelial crypt niche involves Hopx/Klf4 and Tcf7l2/Hnf4‐α cascades. Tissue Barriers, 4, e1214038. 27583195 10.1080/21688370.2016.1214038 PMC4993572

[joa13844-bib-0023] Lin, C.C. , Yao, C.Y. , Hsu, Y.C. , Hou, H.A. , Yuan, C.T. , Li, Y.H. et al. (2020) Knock‐out of Hopx disrupts stemness and quiescence of hematopoietic stem cells in mice. Oncogene, 39, 5112–5123. 32533098 10.1038/s41388-020-1340-2

[joa13844-bib-0024] Liu, Y. & Zhang, W. (2020) The role of HOPX in normal tissues and tumor progression. Bioscience Reports, 40, BSR20191953. 31934721 10.1042/BSR20191953 PMC6997107

[joa13844-bib-0025] Matsumoto, N. , Tanaka, S. , Horiike, T. , Shinmyo, Y. & Kawasaki, H. (2020) A discrete subtype of neural progenitor crucial for cortical folding in the gyrencephalic mammalian brain. eLife, 9, e54873. 32312384 10.7554/eLife.54873 PMC7173966

[joa13844-bib-0026] Molnár, Z. , Clowry, G.J. , Šestan, N. , Alzu'bi, A. , Bakken, T. , Hevner, R.F. et al. (2019) New insights into the development of the human cerebral cortex. Journal of Anatomy, 235, 432–451. 31373394 10.1111/joa.13055 PMC6704245

[joa13844-bib-0027] Nowakowski, T.J. , Bhaduri, A. , Pollen, A.A. , Alvarado, B. , Mostajo‐Radji, M.A. , DI Lullo, E. et al. (2017) Spatiotemporal gene expression trajectories reveal developmental hierarchies of the human cortex. Science, 358, 1318–1323. 29217575 10.1126/science.aap8809 PMC5991609

[joa13844-bib-0028] Nowakowski, T.J. , Pollen, A.A. , Sandoval‐Espinosa, C. & Kriegstein, A.R. (2016) Transformation of the radial glia scaffold demarcates two stages of human cerebral cortex development. Neuron, 91, 1219–1227. 27657449 10.1016/j.neuron.2016.09.005 PMC5087333

[joa13844-bib-0029] Pollen, A.A. , Nowakowski, T.J. , Chen, J. , Retallack, H. , Sandoval‐Espinosa, C. , Nicholas, C.R. et al. (2015) Molecular identity of human outer radial glia during cortical development. Cell, 163, 55–67. 26406371 10.1016/j.cell.2015.09.004 PMC4583716

[joa13844-bib-0030] Rash, B.G. , Duque, A. , Morozov, Y.M. , Arellano, J.I. , Micali, N. & Rakic, P. (2019) Gliogenesis in the outer subventricular zone promotes enlargement and gyrification of the primate cerebrum. Proceedings of the National Academy of Sciences of the United States of America, 116, 7089–7094. 30894491 10.1073/pnas.1822169116 PMC6452694

[joa13844-bib-0031] Scalise, A.A. , Kakogiannos, N. , Zanardi, F. , Iannelli, F. & Giannotta, M. (2021) The blood‐brain and gut‐vascular barriers: from the perspective of claudins. Tissue Barriers, 9, 1926190. 34152937 10.1080/21688370.2021.1926190 PMC8489939

[joa13844-bib-0032] Schmechel, D.E. & Rakic, P. (1979) A Golgi study of radial glial cells in developing monkey telencephalon: morphogenesis and transformation into astrocytes. Anatomy and Embryology., 156, 115–152. 111580 10.1007/BF00300010

[joa13844-bib-0033] Smart, I.H. , Dehay, C. , Giroud, P. , Berland, M. & Kennedy, H. (2002) Unique morphological features of the proliferative zones and postmitotic compartments of the neural epithelium giving rise to striate and extrastriate cortex in the monkey. Cerebral Cortex, 12, 37–53. 11734531 10.1093/cercor/12.1.37 PMC1931430

[joa13844-bib-0034] Stagaard Janas, M. , Nowakowski, R.S. , Terkelsen, O.B. & Møllgård, K. (1991) Glial cell differentiation in neuron‐free and neuron‐rich regions. I. Selective appearance of S‐100 protein in radial glial cells of the hippocampal fimbria in human fetuses. Anatomy and Embryology., 184, 549–558. 1776701 10.1007/BF00942577

[joa13844-bib-0035] Stagaard, M. & Møllgård, K. (1989) The developing neuroepithelium in human embryonic and fetal brain studied with vimentin‐immunocytochemistry. Anatomy and Embryology, 180, 17–28. 2476946 10.1007/BF00321896

[joa13844-bib-0036] Subramanian, L. , Calcagnotto, M.E. & Paredes, M.F. (2020) Cortical malformations: lessons in human brain development. Frontiers in Cellular Neuroscience, 13, 576. 32038172 10.3389/fncel.2019.00576 PMC6993122

[joa13844-bib-0037] Vaid, S. , Camp, J.G. , Hersemann, L. , Eugster Oegema, C. , Heninger, A.K. , Winkler, S. et al. (2018) A novel population of Hopx‐dependent basal radial glial cells in the developing mouse neocortex. Development, 145, dev169276. 30266827 10.1242/dev.169276

[joa13844-bib-0038] Vasistha, N.A. , García‐Moreno, F. , Arora, S. , Cheung, A.F. , Arnold, S.J. , Robertson, E.J. et al. (2015) Cortical and clonal contribution of Tbr2 expressing progenitors in the developing mouse brain. Cerebral Cortex, 25, 3290–3302. 24927931 10.1093/cercor/bhu125 PMC4585488

[joa13844-bib-0039] Yachnis, A.T. & Rorke, L.B. (1999) Cerebellar and brainstem development: an overview in relation to Joubert syndrome. Journal of Child Neurology, 14, 570–573. 10488901 10.1177/088307389901400904

[joa13844-bib-0040] Zec, N. , Rowitch, D.H. , Bitgood, M.J. & Kinney, H.C. (1997) Expression of the homeobox‐containing genes EN1 and EN2 in human fetal midgestational medulla and cerebellum. Journal of Neuropathology and Experimental Neurology, 56, 236–242. 9056537 10.1097/00005072-199703000-00002

[joa13844-bib-0041] Zhong, S. , Ding, W. , Sun, L. , Lu, Y. , Dong, H. , Fan, X. et al. (2020) Decoding the development of the human hippocampus. Nature, 577, 531–536. 31942070 10.1038/s41586-019-1917-5

[joa13844-bib-0042] Žunić Išasegi, I. , Radoš, M. , Krsnik, Ž. , Radoš, M. , Benjak, V. & Kostović, I. (2018) Interactive histogenesis of axonal strata and proliferative zones in the human fetal cerebral wall. Brain Structure & Function, 223, 3919–3943. 30094607 10.1007/s00429-018-1721-2 PMC6267252

[joa13844-bib-0043] Zweifel, S. , Marcy, G. , Lo Guidice, Q. , Li, D. , Heinrich, C. , Azim, K. et al. (2018) HOPX defines heterogeneity of postnatal subventricular zone neural stem cells. Stem Cell Reports, 11, 770–783. 30174314 10.1016/j.stemcr.2018.08.006 PMC6135899

