# Supplementary material for: The HOPX and BLBP landscape and gliogenic regions in developing human brain
Source: J Anat. 2023 Feb 16;243(1):23–38. doi: 10.1111/joa.13844 (PMC10273337; doi:10.1111/joa.13844)
Supplement: Supplementary file 1 — Figure S1 Figure S2 Figure S3 Figure S4 Figure S5 Table S1 [file JOA-243-23-s001.pdf]

Supplementary data

Supplementary figure S1-S5 are stained with H&E in **A** and for GFAP (green) and Syto13 (blue) in **B.** Location of regions of interest (ROIs) are indicated with white circles and squares in **B**. White squares depict free-hand drawn ROIs, which do not include the entire square and are defined in white along with circular ROIs in higher magnification in **C**.

.

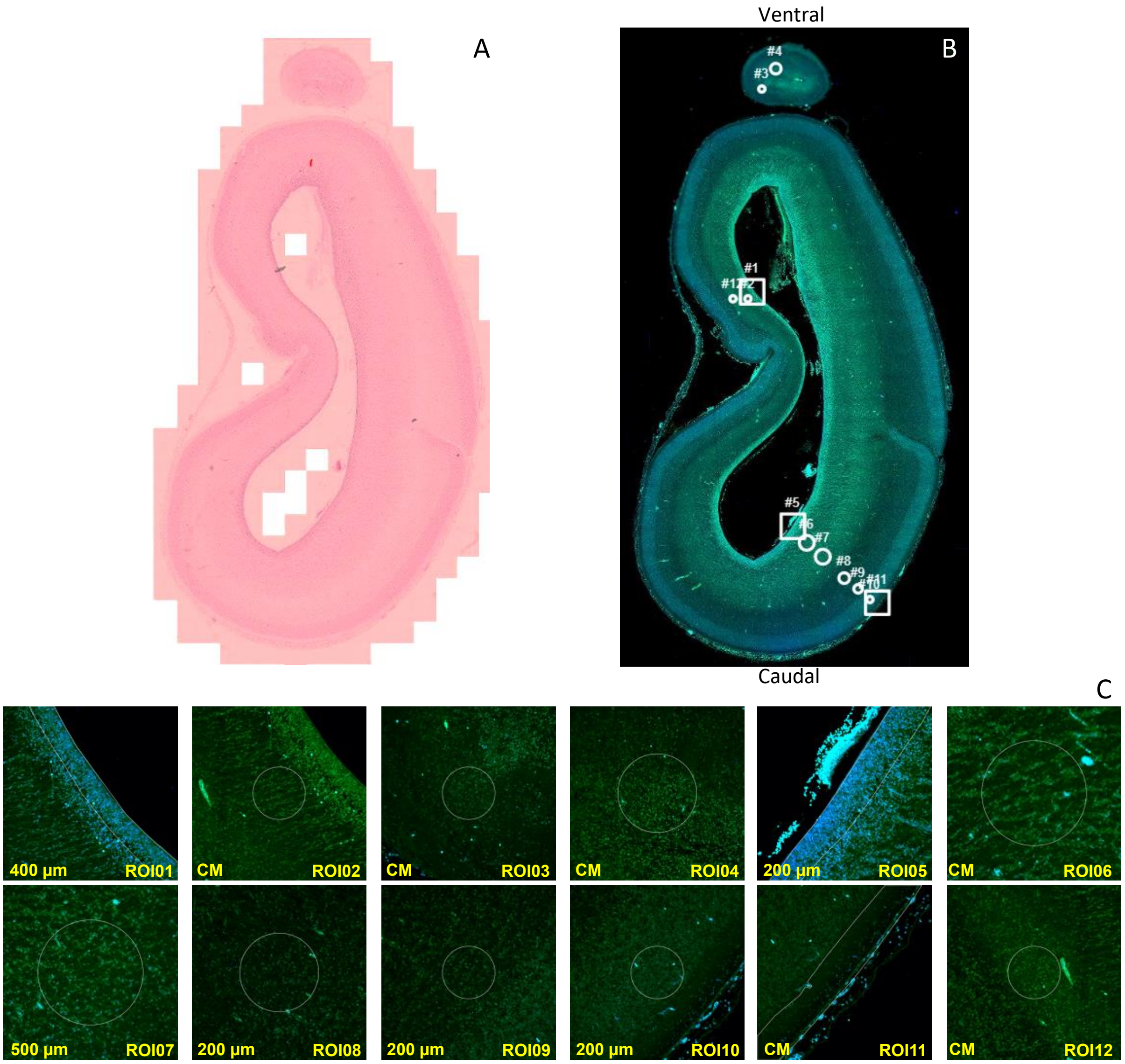

Supplementary figure S1. Coronal section of frontal lobe from a 13 wpc human fetus used for Digital Spatial Profiling (DSP) with Nanostring GeoMx®. Note, that sections shown in **A** and **B** are turned upside-down for technical reasons. ROI-1, VZ/ISVZ (medial wall); ROI-2, IFL (medial wall); ROI-3, olfactory bulb, zone below olfactory nerve layer; ROI-4, central core of olfactory bulb; ROI-5, VZ; ROI-6, IFL; ROI-7: OSVZ; ROI-8, SP; ROI-9, SP; ROI-10, CP; ROI-11, MZ; ROI-12, OSVZ (medial wall). ROIs used for figure 5: ROI-3, ROI-5 to ROI-11.

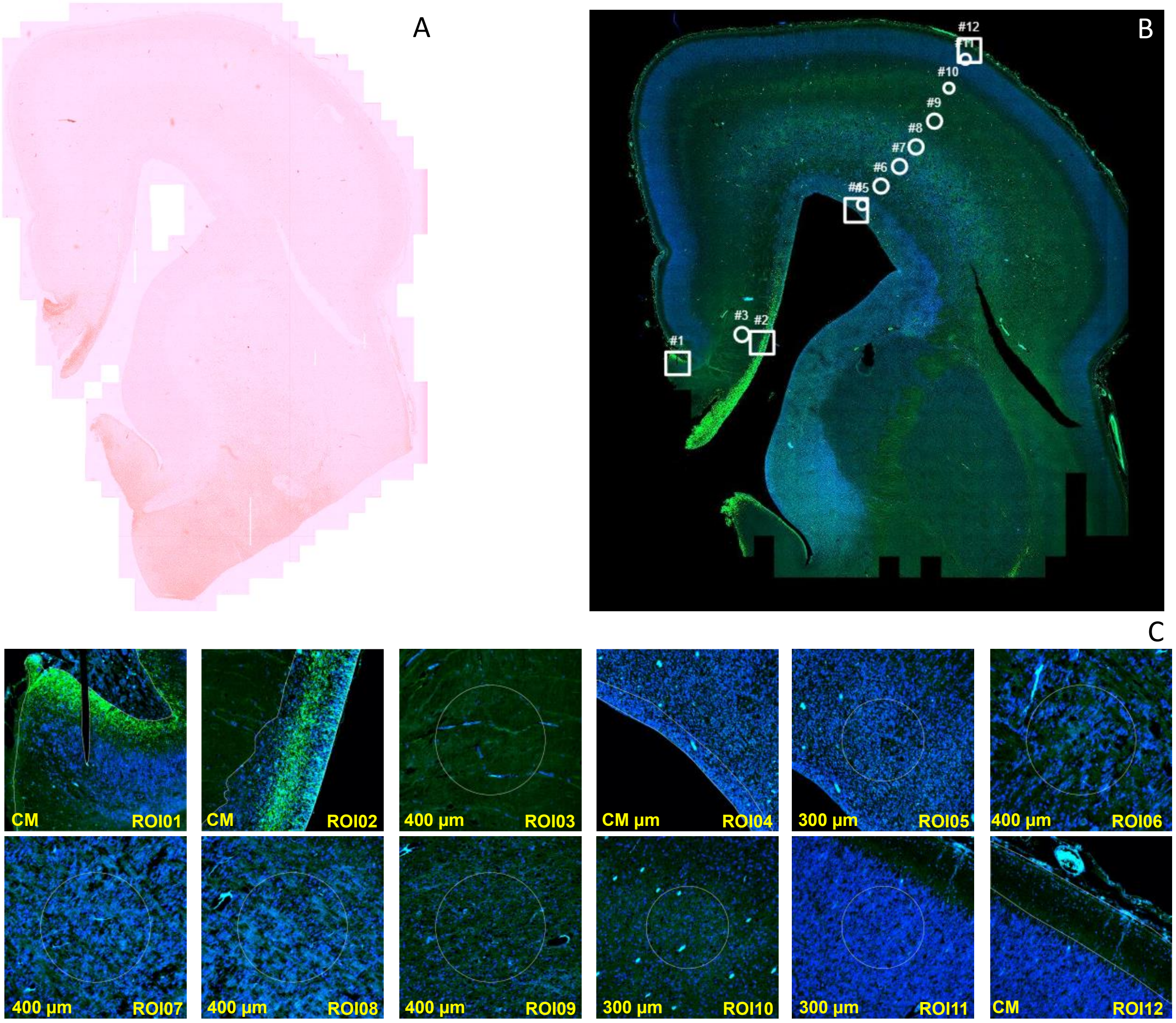

Supplementary figure S2. Coronal section of parietal lobe from a 19 wpc human fetus used for Digital Spatial Profiling (DSP) with Nanostring GeoMx®. ROI-1, indusium griseum; ROI-2, VZ/ISVZ (gyrus cinguli); ROI-3, cingulum; ROI-4, VZ; ROI-5, ISVZ; ROI-6, IFL; ROI-7, OSVZ; ROI-8, OSVZ; ROI-9, SP; ROI-10, SP; ROI-11, CP; ROI-12, MZ. ROIs used for figure 5: ROI-1 to ROI-5, ROI-8 to ROI-12.

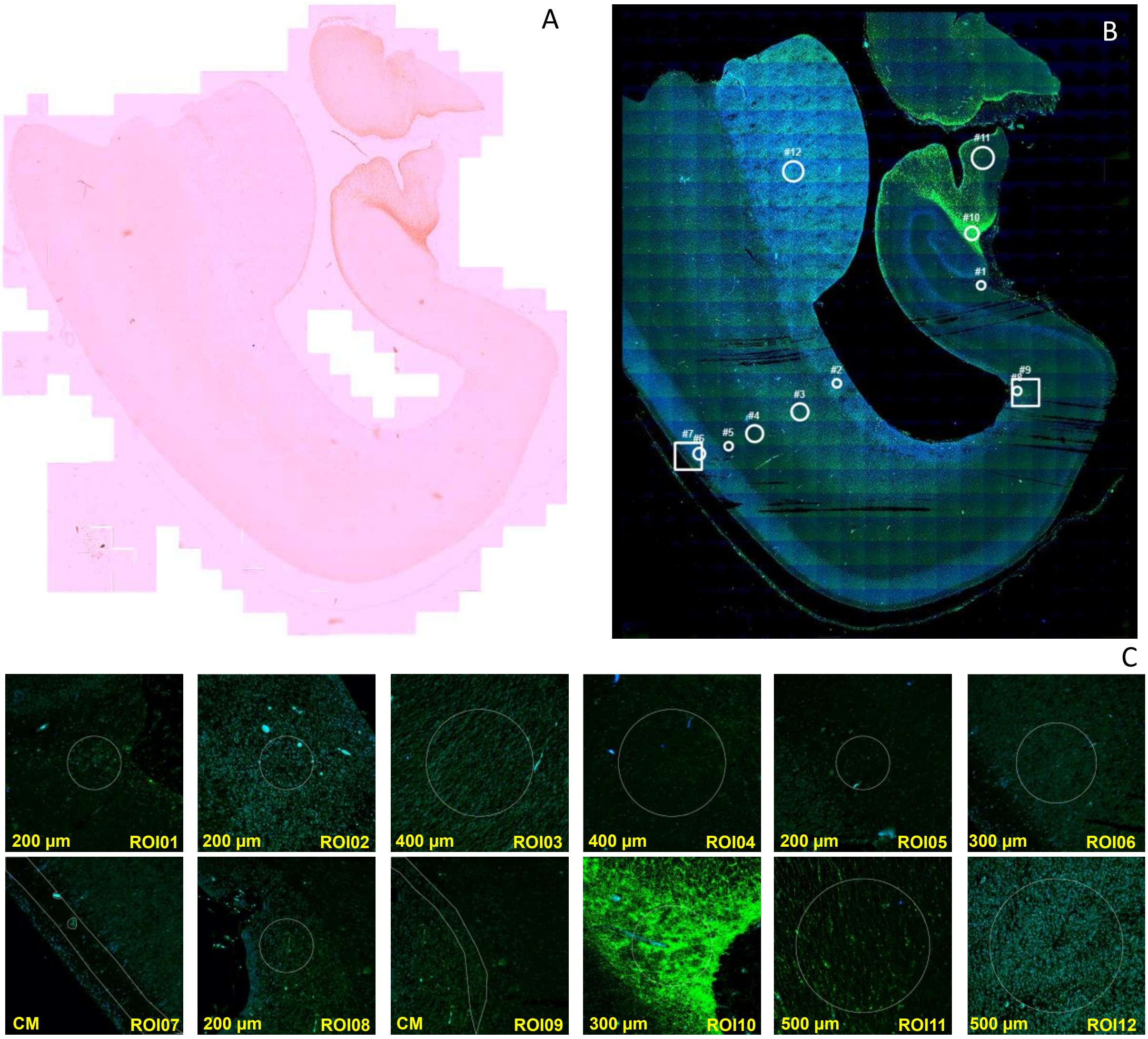

Supplementary figure S3. Coronal section temporal lobe including hippocampus from a 19 wpc human fetus used for Digital Spatial Profiling (DSP) with Nanostring GeoMx®. ROI-1, hippocampal fissure; ROI-2, ISVZ; ROI-3, OSVZ; ROI-4, SP; ROI-5, OSP; ROI-6, CP; ROI-7: MZ; ROI-8, SVZ (entorhinal cortex); ROI-9, SVZ (entorhinal cortex); ROI-10, fimbrio-dentate junction; ROI-11, fimbria; ROI-12, ganglionic eminence. ROIs used for figure 5: ROI-1 to ROI-7, ROI-10 to ROI-11.

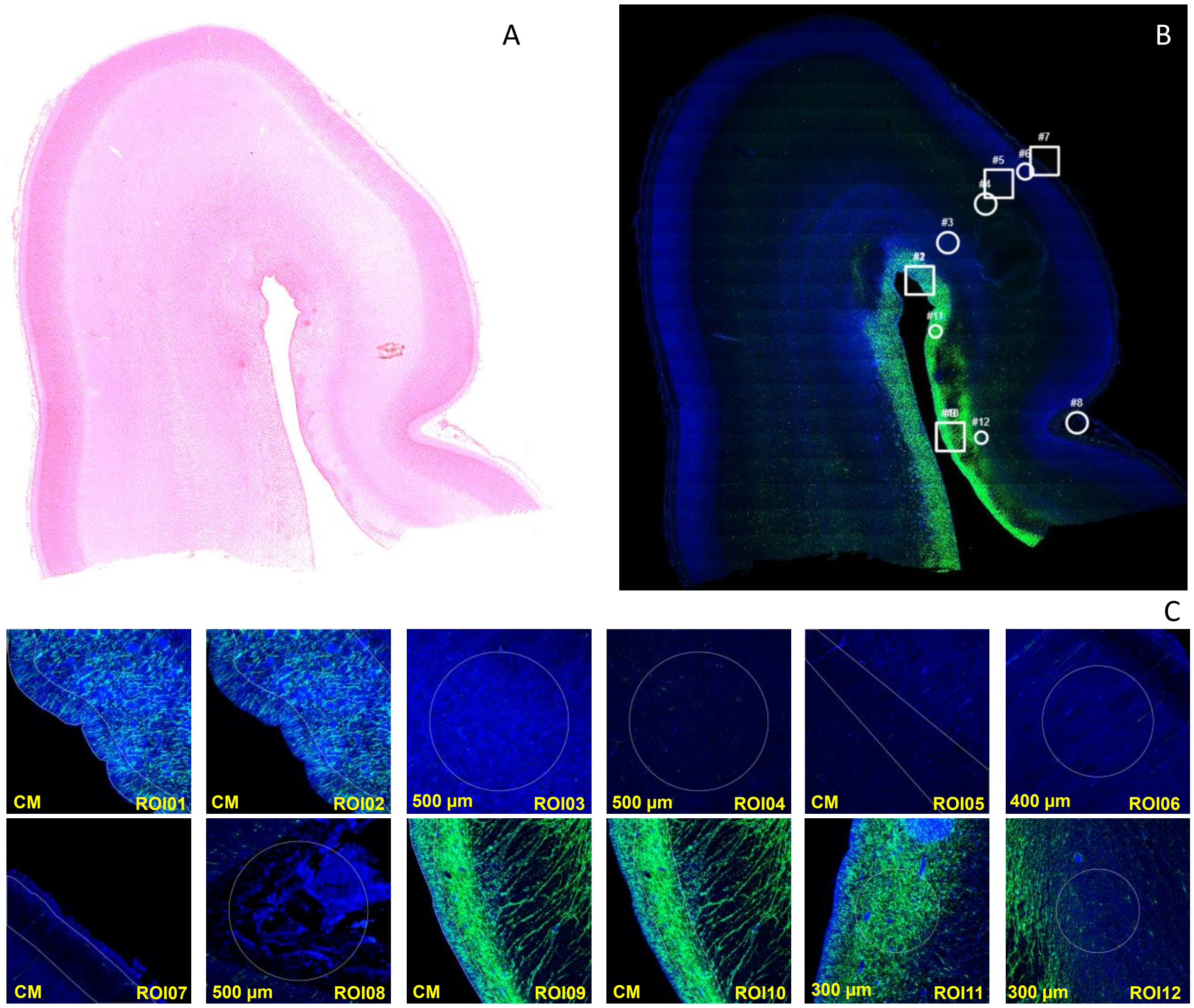

Supplementary figure S4. Coronal section of occipital lobe from a 21 wpc human fetus used for Digital Spatial Profiling (DSP) with Nanostring GeoMx®. ROI-1, VZ; ROI-2, ISVZ; ROI-3, OSVZ; ROI-4, SP; ROI-5, OSP; ROI-6, CP; ROI-7, MZ; ROI-8, Meninges; ROI-9, VZ beneath sulcus calcarinus; ROI-10, SVZ/IFL beneath sulcus calcarinus; ROI-11, ISVZ; ROI-12, condensed OSVZ. ROIs used for figure 5: ROI-1 to ROI-4, ROI-6 to ROI-8.

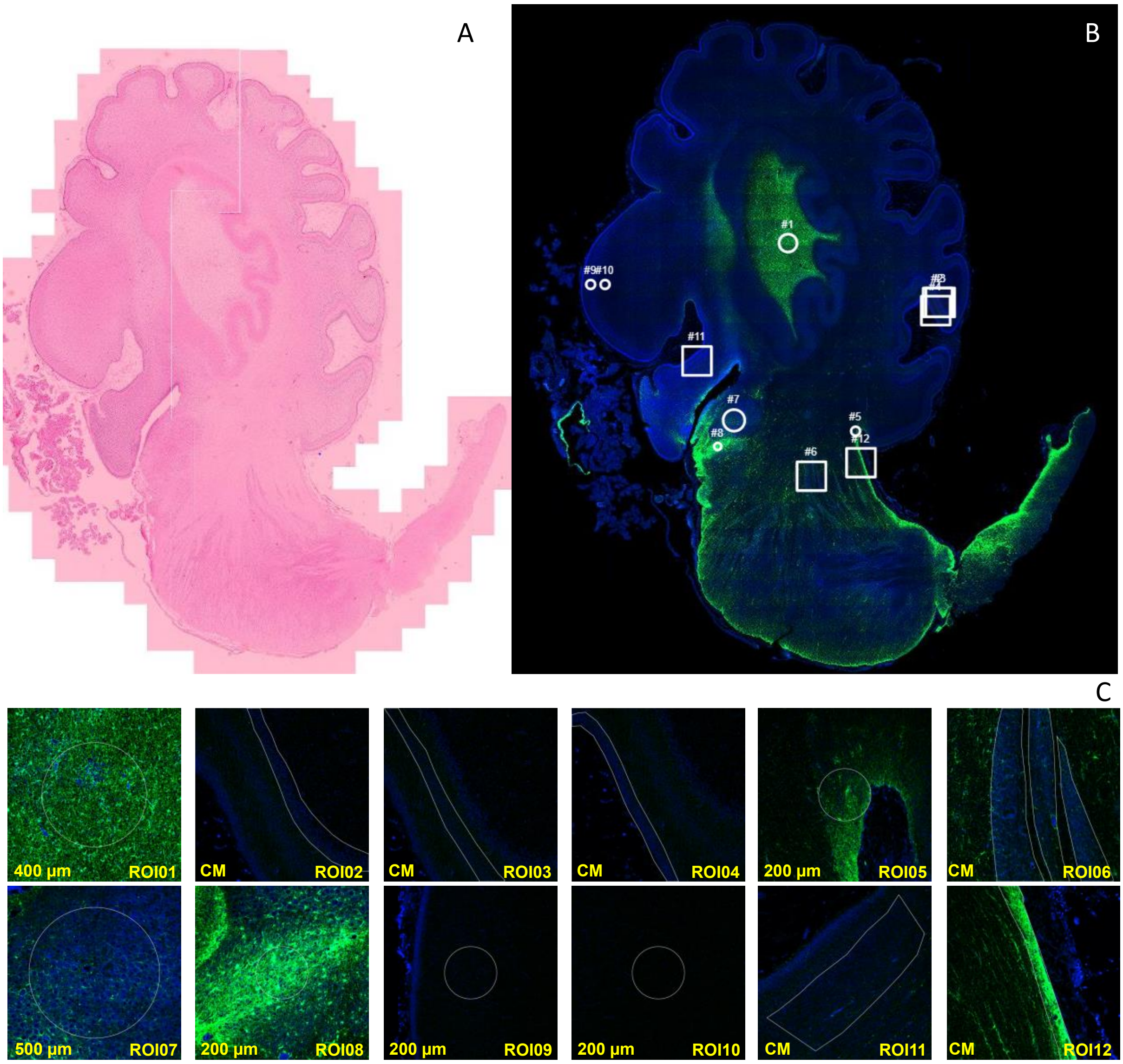

Supplementary figure S5. Sagittal section of brainstem and cerebellum from a 21 wpc human fetus used for Digital Spatial Profiling (DSP) with Nanostring GeoMx®. ROI-1, center of dentate nucleus; ROI-2, IGL; ROI-3, PCL; ROI-4, EGL; ROI-5, hook (gliogenic region shown in Holst et al. figure 8 (Holst et al. J Anat 2019, 235(3):590-615); ROI-6, dense fiber bundles; ROI-7, corpus pontobulbare (central neuronal region); ROI-8, corpus pontobulbare (gliogenic rostral extension); ROI-9, early developing cerebellar cortex; ROI-10, early developing cerebellar cortex (deeper cortical layer); ROI-11, Purkinje cell plate/GL; ROI-12, rostral extension of 'the hook'. ROIs used for figure 5: ROI-1 to ROI-4, ROI-6 to ROI-8.

Table S1. Regions of interest (ROI) used for heatmaps in figure 5.

	Frontal (S1)	Parietal (S2)	Temporal (S3)	Occipital (S4)	Brainstem and cerebellum
Meninges				ROI-8	
Marginal zone (MZ)	ROI-11	ROI-12	ROI-7	ROI-7	
Cortical plate (CP)	ROI-10	ROI-11	ROI-6	ROI-6	
Subplate (SP)	ROI-9	ROI-10	ROI-5	ROI-5 (OBS)	
Subplate (SP)	ROI-8	ROI-9	ROI-4		
Outer subventricular zone (OSVZ)	ROI-7	ROI-8	ROI-3	ROI-3	
Inner subventricular zone (ISVZ)/ Inner fibrous layer (IFL)*	ROI-6* (OBS)	ROI-5	ROI-2	ROI-2	
Ventricular zone (VZ)	ROI-5	ROI-4		ROI-1	
Olfactory bulb (OB)	ROI-3				
Indusium griseum (IG)		ROI-1			
Cingulum		ROI-3			
VZ/ISVZ (gyrus cinguli)		ROI-2			
Hippocampal fissure			ROI-1		
Fimbrio-dentate junction (FDJ)			ROI-10		
Fimbria			ROI-11		
External granular/germinal layer (EGL)					ROI-4
Purkinje cell layer (PCL)					ROI-3
Internal granular layer (IGL)					ROI-2
Dentate nucleus					ROI-1
Fibers					ROI-6
Corpus pontobulbare (neuronal)					ROI-7
Corpus pontobulbare (gliogenic)					ROI-8

*In frontal cortex IFL and not ISVZ were used as ROI.
